# Supplementary material for: Overt hepatic encephalopathy after elective and preemptive TIPS: Risk factors and prognosis
Source: JHEP Rep. 2025 Aug 11;7(11):101548. doi: 10.1016/j.jhepr.2025.101548 (PMC12557589; doi:10.1016/j.jhepr.2025.101548)
Supplement: Multimedia component 1 [file mmc1.pdf]

Overt hepatic encephalopathy after elective or preemptive TIPS: risk factors and prognosis

Marika Rudler, Charlotte Bouzbib, Philippe Sultanik, Charles Roux, Paul Primard, Mélisande Jorus, Lyes Kheloufi, Nicolas Weiss, Asier Rabasco Meneghetti, Benjamin Poussot, Hélène Larrue, Christophe Bureau, José Ursic Bedoya, Sarah Mouri, Dominique Thabut

Table of contents

Fig. S1……………………………………………………………………………………2

Fig. S2……………………………………………………………………………………2

Fig. S3……………………………………………………………………………………3

Fig. S4……………………………………………………………………………………3

Table S1………………………………………………………………………………...4

Table S2………………………………………………………………………………...5

Table S3………………………………………………………………………………...6

Table S4………………………………………………………………………………...8

Table S5……………………………………………………………………………….10

Table S6……………………………………………………………………………….10

Fig. S1.

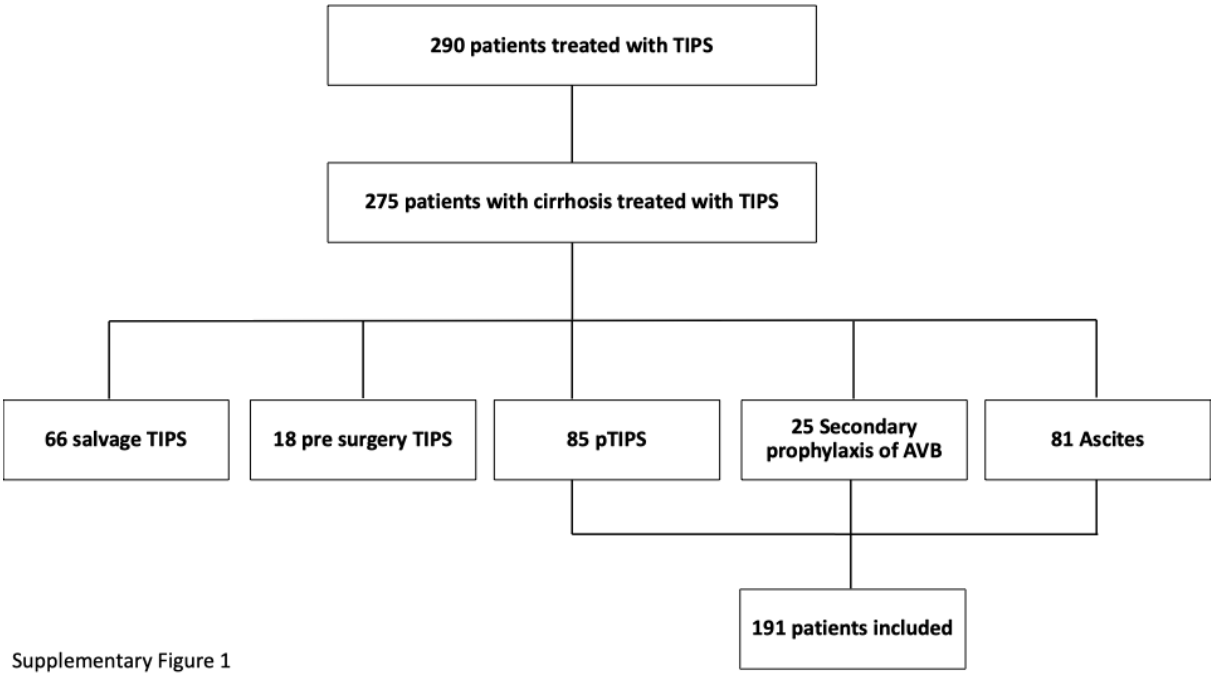

Supplementary Figure 1

Fig. S2.

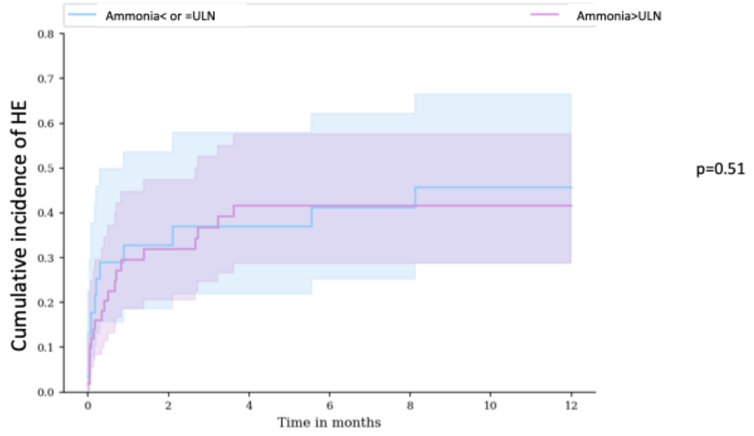

			0	2	4	6	8	10	12
Ammonia< or =ULN	No								
		At risk	27	16	15	14	13	11	0
		Censored	0	3	3	3	4	5	16
		Events	1	9	10	11	11	12	12
Ammonia>ULN	Yes								
		At risk	49	28	22	21	19	18	0
		Censored	1	8	10	11	13	14	32
		Events	1	15	19	19	19	19	19

Supplementary Figure 2

Fig. S3.

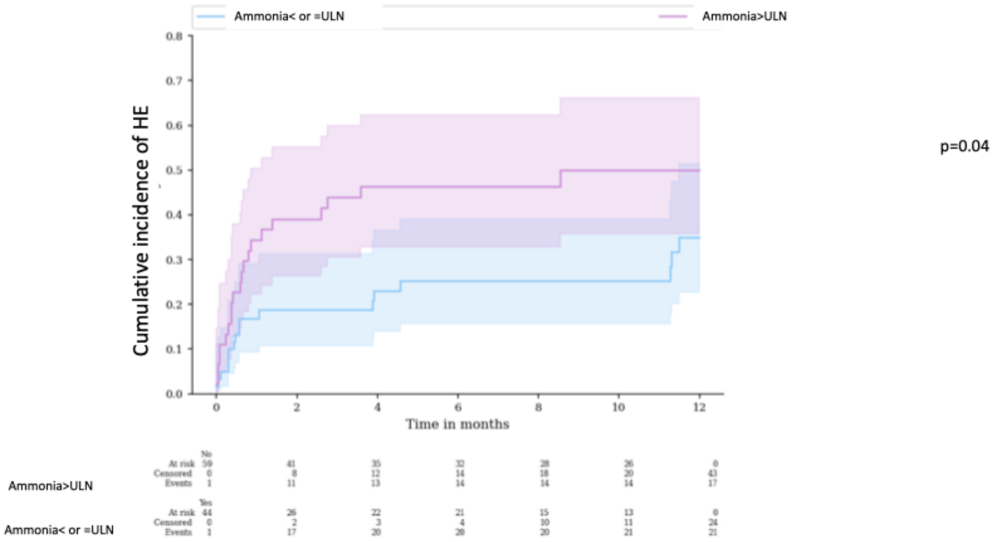

Supplementary Figure 3

Fig. S4.

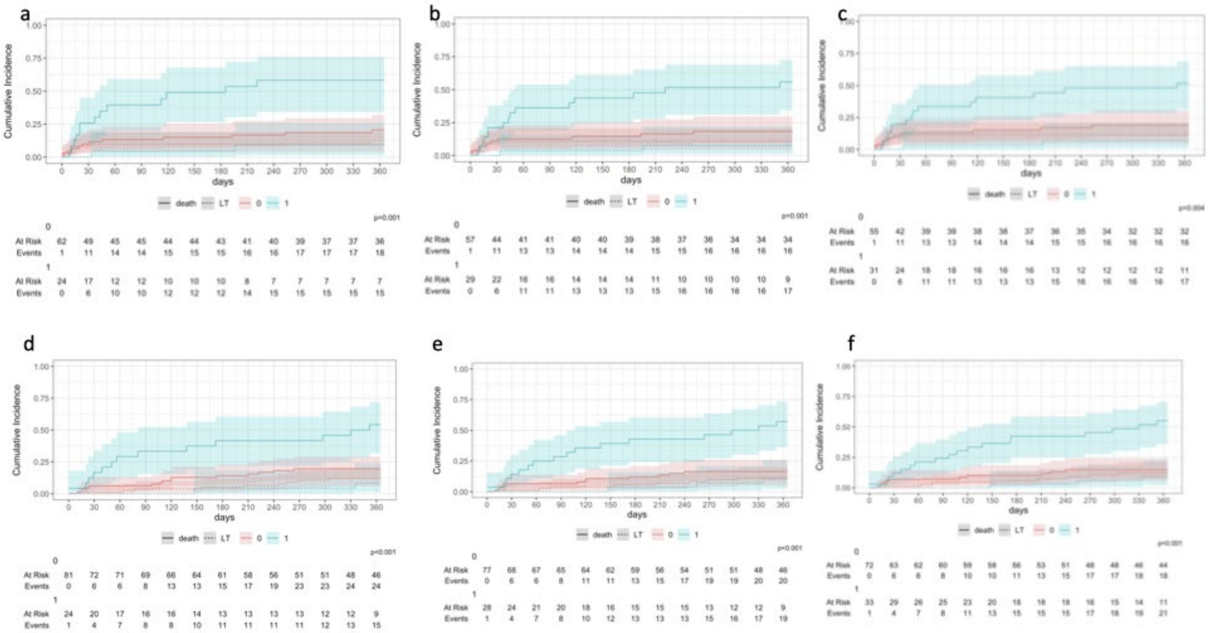

Supplementary Figure 4

Table S1: Baseline Characteristics of patients treated with elective TIPS according to TIPS indication

Variable	Ascites/hydrothorax (n=80)	AVB/surgery (n=26)	p value
Age (years)	59 (57-61)	60 (56-64)	0.82
**Male Gender, n (%)**	63 (78.7)	20 (76.9)	0.99
**BMI (kg/m^2)**	26 (23-30)	25 (23-29)	0.25
**Obesity/overweight (%)**	48 (60)	13 (48)	0.44
**Type 2 diabetes, n (%)**	30 (38)	10 (38)	0.99
**Previous cardiac disease, n (%)**	17 (21.8)	5 (19.2)	0.99
**Previous OHE, n (%)**	18 (22.8)	5 (19.2)	0.80
**Child-Pugh score**	8 (7-10)	7 (5-11)	0.06
**MELD score**	12 (7-18)	11 (8-16)	0.40
**INR**	1.8 (1.6-2.1)	1.7 (1.5-2.2)	0.81
**Serum sodium (mmol/L)**	133 (128-142)	137 (130-144)	<0.001
**Albumin (g/L)**	31 (29-32)	28 (26-29)	0.01
**Bilirubin (μmol/L)**	22 (8-75)	23 (7-72)	0.93
**Ammonia (μmol/L)**	52 (20-180)	59 (16-130)	0.23
**HVPG (mmHg)**	16 (10-25)	17 (11-26)	0.68
**PPG (mmHg)**	7 (6-9)	6 (5-9)	0.47

Group comparisons of categorical variables were performed using Chi-squared test. A p value <0.05 was considered significant.
BMI, body mass index; OHE, overt hepatic encephalopathy; MELD, Model for End-Stage Liver Disease; INR, international normalized ratio; HVPG, hepatic venous pressure gradient; PPG, portal pressure gradient

Table S2: baseline characteristics of patients treated with elective TIPS in the validation cohort and in the study cohort

Variable	Cohort of validation (n=132)	Study cohort (n=106)	p value
Age (years)	59±9	60±9	0.89
**Male Gender, n (%)**	111 (84.1)	26 (78.3)	0.33
**BMI (kg/m^2)**	25±6	26±6	0.25
**Previous OHE, n (%)**	21 (15.9)	23 (21.7)	0.33
**Child-Pugh score**	8±1	8±1	0.10
**MELD score**	13±4	12±3	0.18
**INR**	1.7±0.3	1.4±0.2	0.24
**Serum sodium (mmol/L)**	134±5	135±4	0.46
**Albumin (g/L)**	32±7	30±5	0.04
**Bilirubin (μmol/L)**	30±28	23±16	0.06
**HVPG (mmHg)**	16±5	16±4	0.93
**PPG (mmHg)**	7±3	7±3	0.85

Group comparisons of categorical variables were performed using Chi-squared test. A p value <0.05 was considered significant.
BMI, body mass index; OHE, overt hepatic encephalopathy; MELD, model for end stage liver disease; INR, international normalized ratio; HVPG, hepatic venous pressure gradient; PPG, portal pressure gradient.

Table S3: comparison of clinical, biological and hemodynamic characteristics of patients with or without OHE occurrence after pTIPS

Variable	No OHE (n=45)	OHE (n=39)	p value
Age (years)	52 (46-60)	54 (45-63)	0.54
**Male Gender, n (%)**	35 (78)	36 (92)	0.08
**BMI (kg/m²)**	26 (23-30)	24 (22-29)	0.21
**Cause of liver disease n, (%)**			
**Other**	7 (16)	7 (18)	0.19
**MASLD**	2 (4)	0 (0)	
**ALD**	23 (51)	15 (38)	
**MetADL**	13 (29)	17 (44)	
**Obesity/overweight (%)**	27 (60)	19 (48)	0.41
**Type 2 diabetes, n (%)**	5 (11)	9 (23)	0.15
**Previous cardiac disease, n (%)**	3 (7)	8 (20)	<0.10
**Previous OHE, n (%)**	8 (18)	10 (25)	0.43
**Child-Pugh Class, n (%)**			
**A**	3 (7)	3 (8)	0.40
**B**	7 (16)	11 (28)	
**C**	34 (77)	25 (64)	
**MELD score**	19 (16-23)	18 (15-23)	0.41
**Platelets count (G/L)**	88 (61-137)	78 (61-104)	0.29
**Hemoglobin (g/dL)**	8.1 (7.3-9.5)	8.4 (7.5-9.1)	0.68
**Lactate**	1.7 (1.4-2.1)	1.6 (1.3-2.1)	0.60
**INR**	1.8 (1.6-2.1)	1.7 (1.5-2.2)	0.81
**Fibrinogen (g/L)**	2.1 (1.8-2.4)	2.1 (1.9-2.3)	0.94
**Serum sodium (mmol/L)**	137 (133-138)	137 (132-139)	0.92
**Albumin (g/L)**	26 (24-29)	26 (23-29)	0.64
**Bilirubin (μmol/L)**	72 (47-129)	48 (30-122)	0.14
**Ammonia (μmol/L)**	60 (45-88)	70 (39-108)	0.76
**HVPG (mmHg)**	14 (13-21)	17 (13-18)	0.66
**PPG (mmHg)**	7 (6-9)	6 (5-9)	0.18
**Decrease of mean PPG gradient (%)**	50 (36-64)	54 (42-64)	0.46
**TIPS diameter**	10 (8-10)	10 (8-10)	0.70
**TIPS dilatation**	10 (8-10)	10 (8-10)	0.85

Group comparisons of categorical variables were performed using Chi-squared test. A p value <0.05 was considered significant.

BMI, body mass index; MASLD, Metabolic-dysfunction Associated Steatotic Liver Disease; ALD, Alcohol related liver disease; MetALD, Metabolic and Alcohol related Liver Disease; OHE, overt hepatic encephalopathy; HCC, hepatocellular carcinoma; AVB, acute variceal bleeding; MELD, Model For End-Stage Liver Disease; PT, prothrombin time ratio; INR, international normalized ratio; AST aspartate aminotransferase; ALT, alanine aminotransferase; NT-proBNP, N-terminal pro b-type natriuretic peptide; Clif-C-ACLF

Table S4: comparison of clinical, biological and hemodynamic characteristics of patients with or without OHE occurrence after elective TIPS

Variable	No OHE (n=65)	OHE (n=41)	p value
Age (years)	60 (52-65)	62 (54-67)	0.36
**Male Gender, n (%)**	52 (80)	31 (76)	0.63
**BMI (kg/m^2)**	25 (22-27)	25 (21-30)	0.48
**Cause of liver disease n, (%)**			
**Other**	12 (19)	9 (22)	0.26
**MASLD**	7 (11)	9 (22)	
**ALD**	27 (41)	14 (34)	
**MetADL**	19 (29)	9 (22)	
**Obesity/overweight (%)**	36 (56)	24 (59)	0.98
**Type 2 diabetes, n (%)**	**19 (30)**	**21 (52)**	**0.04**
**Previous cardiac disease, n (%)**	**6 (10)**	**16 (39)**	**<0.001**
**Ischemic cardiopathy, n (%)**	**2 (3)**	**7 (17)**	**0.03**
**Atrial fibrillation, n (%)**	**1 (2)**	**5 (12)**	**0.03**
**Previous OHE, n (%)**	16 (25)	7 (17)	0.47
**Child-Pugh Class, n (%)**			
**A**	6 (9)	3 (7)	0.93
**B**	53 (83)	34 (83)	
**C**	5 (8)	4 (10)	
**MELD score**	11 (10-14)	12 (10-15)	0.34
**Platelets count (G/L)**	**124 (86-168)**	**95 (67-141)**	**0.04**
**Hemoglobin (g/dL)**	9.8 (8.5-11.5)	9.7 (8.4-12.4)	0.40
**Lactate**	1.3 (1.2-1.5)	1.3 (1.2-1.5)	0.98
**INR**	1.5 (1.1-1.8)	1.6 (1.4-1.9)	0.22
**Fibrinogen (g/L)**	3.2 (2.4-3.9)	3.0 (2.2-3.3)	0.17
**Serum sodium (mmol/L)**	136 (131-138)	134 (132-138)	0.97
**Albumin (g/L)**	**32 (28-34)**	**28 (25-31)**	**0.02**
**Bilirubin (μmol/L)**	17 (11-27)	22 (11-33)	0.23
**Ammonia (μmol/L)**	**40 (28-60)**	**55 (37-96)**	**0.01**
**Rifaximin n %**	**39 (61)**	**15 (36)**	**0.02**
**HVPG (mmHg)**	16 (14-19)	16 (14-20)	0.72
**PPG (mmHg)**	6 (5-8)	7 (4-8)	0.84

**Decrease of mean PPG gradient (%)**	58 (50-67)	63 (53-71)	0.38
**TIPS diameter**	8 (8-10)	10 (8-10)	0.08
**TIPS dilatation**	8 (8-10)	8 (8-10)	0.21

Group comparisons of categorical variables were performed using Chi-squared test. A p value <0.05 was considered significant.
BMI, body mass index; MASLD, Metabolic-dysfunction Associated Steatotic Liver Disease; ALD, Alcohol related liver disease; MetALD, Metabolic and Alcohol related Liver Disease; OHE, overt hepatic encephalopathy; HCC, hepatocellular carcinoma; AVB, acute variceal bleeding; MELD, Model For End-Stage Liver Disease; PT, prothrombin time ratio; INR, international normalized ratio; AST aspartate aminotransferase; ALT, alanine aminotransferase; NT-proBNP, N-terminal pro b-type natriuretic peptide; Clif-C-ACLF

Table S5: Univariate and multivariate analysis of factors associated with OHE after elective TIPS in the validation cohort

	Univariate analysis for OHE in the elective TIPS group (validation cohort) HR [95% CI], p-value	p	Multivariate analysis for OHE in the elective TIPS group (validation cohort) HR [95% CI], p-value	p
**Age**	1.03 [1.00-1.06]	0.04	1.03 [0.99-1.07]	0.07
**Previous OHE**	1.93 [0.98-3.80]	0.06	1.91 [0.95-3.82]	0.07
**Platelets count**	1.00 [0.99-1.00]	0.17		
**Serum sodium**	0.97 [0.92-1.02]	0.23		
**Albumin**	0.96 [0.91-1.01]	0.09	0.97 [0.92-1.02]	0.23
**Creatinine**	**1.01 [1.00-1.01]**	**0.03**	**1.01 [1.00-1.01]**	**0.04**
**MELD score**	1.03 [0.97- 1.10]	0.35		
**PPG**	0.98 [0.90-1.07]	0.67		

Bolded values indicate statistically significant differences (p < 0.05) (Fine and Gray models).

OHE, overt hepatic encephalopathy; MELD, model for end stage liver disease; PPG, portal pressure gradient.

Table S6: outcomes of patients treated with elective TIPS according to TIPS indication

Outcome	Ascites/hydrothorax (n=80)	AVB/surgery (n=26)	p value
**Death n (%)**	**29 (36.2)**	**6 (23.0)**	**0.24**
**LT**	**16 (20.0)**	**2 (7.7)**	**0.23**
**OHE**	**31 (38.8)**	**10 (38.5)**	**0.99**

LT, liver transplantation; OHE, overt hepatic encephalopathy;
