# Supplementary material for: Overt hepatic encephalopathy after elective and preemptive TIPS: Risk factors and prognosis
Source: JHEP Rep. 2025 Aug 11;7(11):101548. doi: 10.1016/j.jhepr.2025.101548 (PMC12557589; doi:10.1016/j.jhepr.2025.101548)
Supplement: Multimedia component 4 [file mmc4.pdf]

Overt hepatic encephalopathy after elective and preemptive TIPS: Risk factors and prognosis

Authors

Marika Rudler, Charlotte Bouzbib, Philippe Sultanik, …, José Ursic Bedoya, Sarah Mouri, Dominique Thabut

Correspondence

marika.rudler@aphp.fr (M. Rudler).

Graphical abstract

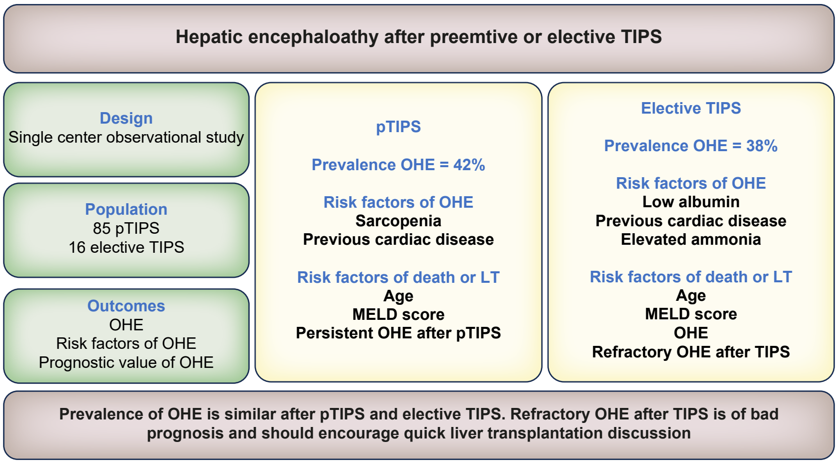

Highlights:

- OHE prevalence was similar after pTIPS and elective TIPS.
- Risk factors are different according to clinical situation.
- Persistent OHE after pTIPS and refractory OHE after elective TIPS are associated with poor outcomes.
- Liver transplantation should be quickly discussed in case of persistent/refractory OHE after TIPS.

Impact and implications:

This study demonstrates that overt hepatic encephalopathy (OHE) remains a frequent complication after both preemptive and elective transjugular intrahepatic portosystemic shunt (TIPS) placement, though the underlying risk factors differ depending on the clinical indication. Identifying predictors such as cardiac disease, sarcopenia, and baseline ammonia can help refine patient selection and post-TIPS management. The association between OHE and adverse outcomes highlights the need for closer surveillance and timely intervention. Early referral for liver transplantation should be considered in patients who develop persistent or refractory OHE after TIPS.

https://doi.org/10.1016/j.jhepr.2025.101548

© 2025 The Author(s). Published by Elsevier B.V. on behalf of European Association for the Study of the Liver (EASL). This is an open access article under the CC BY license (http://creativecommons.org/licenses/by/4.0/).

JHEP|Reports

Overt hepatic encephalopathy after elective and preemptive TIPS: Risk factors and prognosis

Marika Rudler[1,2,3,*], Charlotte Bouzbib[1,3], Philippe Sultanik[1,3], Charles Roux[4], Paul Primard[1], Mélisande Jorus[1], Lyes Kheloufi[1,3], Nicolas Weiss[2,3,5], Asier Rabasco Meneghetti[6], Benjamin Poussot[4], Hélène Larrue[7], Christophe Bureau[7], José Ursic Bedoya[8], Sarah Mouri[1,3], Dominique Thabut[1,2,3]

JHEP Reports **2025**. vol. 7 | 1–10

Check for updates

Background & Aims: Overt hepatic encephalopathy (OHE) develops in 30-50% of patients after transjugular intrahepatic portosystemic shunt (TIPS) placement, depending on patient characteristics and TIPS indication. Data on OHE after preemptive TIPS (pTIPS) are limited. We aimed to assess the prevalence of OHE after pTIPS, to compare OHE after pTIPS and elective TIPS, and to identify risk factors for OHE in each clinical situation.

Methods: We performed a single-center observational study of consecutive patients with cirrhosis treated with pTIPS or elective TIPS between 2017 and 2023. Patients were followed until 1 year, death, or liver transplantation (LT).

Results: A total of 191 patients were included (pTIPS 85, elective TIPS 106). Patients treated with pTIPS were significantly younger (53 *vs.* 60 years old, p <0.001), had a higher MELD score (20 *vs.* 12, p <0.001), and a higher baseline ammonia level (76 *vs.* 54 μmol/L, p <0.001) compared to those treated with elective TIPS. OHE occurred in 41% of patients undergoing pTIPS compared to 38% of those receiving elective TIPS (p = 0.6). In multivariate analysis, factors associated with the development of OHE in patients undergoing pTIPS were previous cardiac disease (p = 0.02) and sarcopenia (p = 0.016), whereas baseline ammonia (p = 0.022), previous cardiac disease (p = 0.009) and albumin (p = 0.018) were associated with OHE occurrence in patients receiving elective TIPS. In pTIPS, age, MELD score and persistent OHE after TIPS were independently associated with death or LT. In elective TIPS, age, MELD score, OHE, and refractory OHE were independently associated with death or LT.

Conclusion: OHE occurs frequently after TIPS, in elective as well as urgent indications. Risk factors for OHE differ in these situations. Persistent OHE after pTIPS and OHE after elective TIPS are associated with poor outcomes and should prompt early discussion of LT candidacy.

© 2025 The Author(s). Published by Elsevier B.V. on behalf of European Association for the Study of the Liver (EASL). This is an open access article under the CC BY license (http://creativecommons.org/licenses/by/4.0/).

Introduction

Transjugular intrahepatic portosystemic shunt (TIPS) has significantly improved the prognosis of patients with cirrhosis and portal hypertension-related complications, increasing liver transplant-free survival, decreasing further decompensation and improving quality of life in all indications[1] compared with “standard of care”. TIPS is indicated mostly in elective settings: for refractory/recurrent ascites and for failure of secondary prophylaxis of variceal bleeding.[2] Other indications include preemptive TIPS (pTIPS), *i.e.* a TIPS placed within 72 h after control of index bleeding, in patients at high risk of rebleeding, and salvage/rescue TIPS in refractory bleeding or early recurrence of bleeding.[3]

Overt hepatic encephalopathy (OHE) represents the most feared side effect of TIPS, with a prevalence ranging from 30% to 50% depending on patient characteristics and TIPS indication.[4–9] OHE, outside the context of TIPS placement, is a severe complication of cirrhosis,[10,11] independently associated with poor survival,[12,13] with 1-year mortality of more than 50%. In the setting of TIPS, several studies have suggested that the prognosis of OHE after TIPS is poor.[9] Nevertheless, the question remains debated, as a recent large study including more than 600 patients showed that episodic OHE did not increase the risk of mortality in patients treated with elective TIPS.[8] The timing of OHE may influence prognosis, with some authors suggesting that early OHE after TIPS could be particularly detrimental.[14]

Although neurological prognostication is difficult in patients before elective TIPS,[15] risk factors for OHE such as age, covert HE, sarcopenia, low portal pressure gradient after TIPS, renal failure and hyponatremia are well established as associated with a higher HE incidence.[9,16–19] Identification of these risk factors and individualized patient selection, in addition to rifaximin prophylaxis, likely represent the best strategy to prevent OHE after elective TIPS.[3,20,21] In contrast, although randomized controlled trials (RCTs) and observational studies have reported an OHE prevalence of 25%–40% after pTIPS, the risk factors for OHE in this setting remain poorly characterized.

* Corresponding author. Address: Hepatology department, Sorbonne Université, La Pitié Salpêtrière Hospital, 75013 Paris, France.
E-mail address: marika.rudler@aphp.fr (M. Rudler).
https://doi.org/10.1016/j.jhepr.2025.101548

Therefore, the objectives of this study were: i) to assess the prevalence of OHE after pTIPS, ii) to compare OHE after pTIPS and elective TIPS for ascites or secondary prophylaxis of acute variceal bleeding, and iii) to identify risk factors for OHE in each clinical scenario.

Patients and methods

Patients

Study cohort

This is a retrospective study using a prospective cohort of patients treated with TIPS and hospitalized in the department of Hepatology in La Pitié-Salpêtrière hospital, Paris, France. This cohort was approved by the research ethics committee of Sorbonne University (CER-2022-074). All consecutive patients treated with TIPS and hospitalized between September 2017 and October 2023 were screened for inclusion after providing their non-opposition, as recorded in a prospective database.

Inclusion criteria were patients with cirrhosis (known or previously undiagnosed, either histologically confirmed or diagnosed based on clinical or radiological criteria) who underwent TIPS placement, with indications including pTIPS, refractory or recurrent ascites, or failure of secondary prophylaxis. Exclusion criteria were age <18 years, previous liver transplantation (LT), rescue TIPS, or other indications for TIPS.

Validation cohort (elective TIPS)

An external validation cohort was used to confirm the results regarding risk factors for OHE after elective TIPS: 132 consecutive patients treated with TIPS for ascites between 2017 and 2022 (Toulouse, Montpellier, Paris) were analyzed.

Collected variables

Patient history collection included prior ascites, OHE, covert HE, acute variceal bleeding (AVB), hepatocellular carcinoma (HCC), hepatorenal syndrome, alcohol-related hepatitis, portal vein thrombosis, esophageal varices with grade, and chronic kidney disease. For each patient, the etiological work-up was detailed, including chronic viral infections (hepatitis B or C), alcohol use (current or previous), metabolic risk factors (current or past obesity, type 2 diabetes, arterial hypertension, dyslipidemia), autoimmune hepatitis, biliary diseases (primary biliary cholangitis or primary sclerosing cholangitis), Wilson's disease, hemochromatosis, and alpha-1-antitrypsin deficiency. Significant comorbidities, such as cardiac diseases, were also recorded.

The clinical and biological data collected at admission included gender, age, ascites, OHE, AVB, current treatment with non-selective beta blockers, hemoglobin, platelet count, leukocytes, prothrombin time ratio and international normalized ratio, bilirubin, lactate, ammonia, creatinine, albumin, serum sodium, aspartate aminotransferase, alanine aminotransferase, gamma-glutamyltransferase. Child-Pugh score, MELD score, and CLIF-ACLF (acute-on-chronic liver failure) score were calculated. Sarcopenia was assessed in patients with available CT scans. Skeletal muscle index was calculated by measuring the cross-sectional area of skeletal muscle at the third lumbar vertebra (L3) on baseline CT scans. Sarcopenia was defined as skeletal muscle index <53 cm^2/m^2 for men and <41 cm^2/m^2 for women. Total shunt area was also evaluated, and shunts were considered large when >8 mm.[22]

Clinical management

pTIPS

High-risk patients with AVB related to esophageal varices were managed according to Baveno VII recommendations:[23] all received vasoactive drugs, antibiotic therapy and band ligation (performed during initial endoscopy within 6 h after admission). pTIPS was placed in the absence of strict contraindication within 72 h after stabilization, and in patients with Child-Pugh B cirrhosis and active bleeding at endoscopy or Child-Pugh C 10-13 cirrhosis.[24] As our study began before the publication of the individual meta-analysis showing the benefit of pTIPS in patients with Child-Pugh score >7,[25] we included patients with Child-Pugh cirrhosis B7 and active bleeding in the current study. Since 2021, systematic treatment with lactulose was started in patients with AVB.[23]

Elective TIPS

Elective TIPS were placed either for refractory or recurrent ascites[25] or failure of secondary prophylaxis for AVB. Absolute contraindications for elective TIPS placement were severe liver failure, heart failure, severe porto-pulmonary hypertension (mean pulmonary artery pressure >45 mmHg at right heart catheterization), severe renal failure (serum creatinine >3 mg/dl), recurrent or persistent OHE despite adequate treatment, uncontrolled sepsis, and HCC beyond Milan criteria. After the publication of the RCT by Bureau *et al.*,[20] all candidates for elective TIPS placement received prophylactic treatment for OHE with rifaximin 550 mg twice a day (starting 2 weeks before TIPS placement and continued for 6 months after).

All patients were discussed by a multidisciplinary liver transplantation team at the time TIPS was considered, as previously described.[26]

TIPS placement

More than 20 procedures were performed each year in the center, except in 2020 due to the COVID-19 pandemic. All patients underwent TIPS placement using volume-controlled 8- or 10-mm stents (W.L. Gore SRL, Flagstaff, AZ) as previously described,[5] dilated to 8 or 10 mm, according to hemodynamic response. The aim was to reduce portal pressure gradient (PPG) below 12 mmHg. Hepatic venous pressure gradient and PPG were evaluated before and immediately after TIPS placement, respectively, under general anesthesia. Systematic embolization of shunts, before or after TIPS placement was not performed in our center.

Follow-up

After discharge, each patient was followed-up at 1 month and then regularly (each 3 months or more if needed) by a hepatologist of the hepato-gastroenterology department of La Pitié-Salpêtrière hospital. Follow-up data (occurrence of OHE [including asterixis alone], further decompensation, LT, death) were collected at each consultation, as well as re-admission.

Definitions

OHE was diagnosed following the European Association for the Study of the Liver Practice guidelines.[21] Only OHE was considered for the study. OHE was graded according to the West Haven classification,[21] OHE was defined as West Haven grade 2-4. Patients with isolated asterixis were considered to have grade 2 OHE, according to the guidelines.

New OHE was defined by the development of OHE during follow-up in patients who were free of OHE at admission. Absence of OHE was defined by the absence of OHE during follow-up in patients who were free of OHE at admission.

Persistent OHE after pTIPS was defined by OHE at admission that did not resolve after pTIPS placement during follow-up.

Refractory OHE after TIPS was defined by OHE occurring after TIPS placement that did not resolve during follow-up.

Resolution of OHE was defined by the absence of OHE (West Haven <2) after an episode of OHE.

Baseline ammonia > the upper limit of normal (ULN) was defined by an ammonia level >50 μmol/L.

Previous history of cardiac disease was defined by either ischemic, rhythmic or valvular cardiac disease. We defined ischemic cardiac disease if the patient was treated with either a coronary artery stent or coronary bypass. Rhythmic cardiopathy was defined by a previous or current episode of atrial fibrillation, atrial flutter or supraventricular tachycardia.

Statistical analyses

Continuous variables were expressed as means ± standard deviations or medians with interquartile ranges, depending on whether they were normally or non-normally distributed. Categorical variables were summarized as frequencies and percentages. Characteristics of patients were compared using chi-squared (for categorical variables) and independent-samples t/Wilcoxon tests (for normally/non-normally distributed continuous variables). Survival rates were calculated using the Kaplan-Meier method and compared using the log-rank test. Hazard ratios (HRs) and 95% CIs were calculated using Cox proportional hazard ratio survival analysis. We performed Gray's test to compare mortality (with liver transplantation as a competing event) or OHE (with liver transplantation and death as competing events) between different groups. Univariate analyses used all available variables. Selected variables associated with the outcome in the univariate analysis (p <0.1) were incorporated into the multivariate analysis, with the number of covariates limited to one per 10 events. Proportional hazards assumption was checked based on smoothed plots of Schoenfeld residuals. Cox regression model and competing risk models (Gray's test) were used to identify independent predictors of death.

All statistical analyses were performed using RStudio software (2023.12.0+369), Posit team (2023). A two-tailed p value <0.05 was considered significant.

Results

Baseline characteristics of the population

Between August 2017 and November 2023, 290 patients were treated with TIPS in our center. Among them, 15 had liver diseases other than cirrhosis. In the subgroup of patients with cirrhosis, the indications for TIPS placement were salvage TIPS in 66 patients, and pre-surgery in 18 patients. Therefore, 191 patients were included in our study (Fig. S1): 85 received pTIPS and 106 elective TIPS (81 for ascites and 25 for secondary prophylaxis of AVB). Baseline characteristics of the whole cohort and according to TIPS indication are depicted in Table 1: 81% were male, mean age was 57 ± 10 years, and mean MELD score was 15 ± 6. Patients treated with pTIPS were significantly younger (53 ± 11 *vs.* 60 ± 9 years, p <0.001), had more alcohol (ALD)-related or metabolic and alcohol-related (MetALD) cirrhosis (81.2 *vs.* 65.1%, p = 0.024), and were less likely to be abstinent (19.5% *vs.* 54.5%, p <0.001). OHE at admission was present in 31/85 (36.5%) patients in the pTIPS group *vs.* 0/106 (0%) in the elective TIPS group (p <0.001). In the pTIPS group, patients had worse liver function, with higher MELD (20 ± 6 *vs.* 12 ± 3, p <0.001) and Child-Pugh (10.2 ± 2.0 *vs.* 8.0 ± 1.2, p <0.001) scores, as well as higher ammonia (76 ± 46 *vs.* 54 ± 36 μmol/L, p <0.001) and bilirubin (100 ± 96 *vs.* 20 ± 16 μmol/L, p <0.001) levels, compared to those treated with elective TIPS. In the elective TIPS group, ascites was present in 84 (80.0%) patients, *vs.* 51 (60.7%) patients of the pTIPS group (p = 0.006). Regarding ammonia-lowering therapies, patients were significantly more frequently treated with lactulose in the pTIPS group (71.8 *vs.* 34.3%, p <0.001), and with rifaximin in the elective group (50.9 *vs.* 11.8%, p <0.001). In the subgroup analysis, when considering patients treated with elective TIPS either for ascites (n = 80) or another cause (AVB or before surgery, n = 26), we did not find any significant differences regarding baseline characteristics (Table S1) except regarding baseline serum sodium and albumin, which were lower in the subgroup of patients receiving TIPS for ascites or hydrothorax (p <0.001). We performed a validation of the results obtained in the elective TIPS group using a validation cohort. Characteristics of the 132 patients of the validation cohort were similar to those of the study cohort except for a slightly higher serum albumin (32 ± 7 *vs.* 30 ± 5, p = 0.04) (Table S2).

Development of OHE and outcomes during follow-up in patients treated with pTIPS

The 1-year cumulative incidence of OHE, considering death or LT as competing events, after pTIPS was 41% (95% CI 30-52%) (Fig. 1). Baseline characteristics of patients treated with pTIPS according to the development of OHE are given in Table S3. The mean delay between pTIPS placement and OHE was 13 [2-82] days. Among 31 patients with OHE at admission, resolution of OHE occurred in 18 patients (58.1%). Among the 13 patients with OHE at admission that did not reverse after TIPS (*i.e.* persistent OHE), 10 patients died within the first month after TIPS (multiorgan failure related to infection in nine patients and COVID-19 in one patient) and three were transplanted for liver failure (Fig. 2).

Factors associated with OHE after pTIPS are depicted in Table 2. In univariate analysis, type 2 diabetes (HR 2.18, 95% CI 1.06-4.50, p = 0.03), previous cardiac disease (HR 2.16, 95% CI 1.03-4.50, p = 0.04), sarcopenia (HR 2.91, 95% CI 1.03-8.27, p = 0.04) and large spontaneous shunts (HR 1.90, 95% CI 0.89-3.47, p = 0.06) were associated with OHE after pTIPS, but not baseline ammonia (HR 1.00, 0.99-1.01, p = 0.74). In multivariate analysis, independent factors that

Table 1. Baseline characteristics of patients treated with TIPS (whole cohort).

Variable	Whole cohort (N = 191)	Elective TIPS (n = 106)	pTIPS (n = 85)	*p* value
Age (years)	57 ± 10	60 ± 9	53 ± 11	**<0.001**
Male gender n (%)	155 (81)	82 (77)	73 (85)	0.314
BMI (kg/m^2)	26 ± 5	26.1 ± 6.0	26.4 ± 7.0	0.46
Obesity/overweight n (%)	106 (56)	60 (57.1)	46 (54.1)	0.787
Cause of liver disease n (%)				**0.024**
MASLD	18 (9)	16 (15.1)	2 (2.3)	
MetALD	58 (31)	28 (26.4)	30 (35.3)	
ALD	80 (42)	41 (38.7)	39 (45.9)	
Virus	21 (11)	12 (11.3)	9 (10.5)	
Other	14 (7)	9 (8.5)	5 (5.9)	
Alcohol abstinence, n (%)	**71 (29)**	**55 (54.5)**	**16 (19.5)**	**<0.001**
Previous hospitalization ICU, n (%)	**69 (36.7)**	**31 (29.2)**	**38 (44.7)**	**0.042**
Dyslipidemia, n (%)	33 (17.4)	21 (19.8)	12 (14.1)	0.383
Hypertension, n (%)	**57 (30.5)**	**41 (38.7)**	**16 (18.8)**	**0.005**
Type 2 diabetes, n (%)	**54 (28.4)**	**40 (38.1)**	**14 (16.4)**	**0.002**
Smoking status (yes, %)	**89 (48.4)**	**40 (39.2)**	**49 (59.7)**	**0.009**
Previous cardiac disease, n (%)	33 (17.5)	22 (21.15)	11 (12.9)	0.198
Previous OHE	41 (21.6)	23 (21.9)	18 (21.18)	0.999
Previous HCC, n (%)	7 (3.7)	4 (3.8)	3 (3.5)	>0.999
Previous AVB, n (%)	71 (37.4)	45 (42.7)	26 (30.6)	0.112
Previous ascites n (%)	**51 (26.7)**	**48 (45.3)**	**3 (3.5)**	**<0.001**
L3SMI (cm^2/m^2)	**48 (10)**	**48 (9)**	**47 (12)**	**0.29**
Sarcopenia (%)	**60.7**	**57**	**65**	**0.50**
Child-Pugh Class A/B/C n*	**15/69/105**	**9/87/9**	**6/18/60**	**<0.001**
Child-Pugh score	**9.0 ± 2.0**	**8.0 ± 1.2**	**10.2 ± 2.0**	**<0.001**
MELD score	**15 ± 6**	**12 ± 3**	**20 ± 6**	**<0.001**
Hemoglobin (g/dl)	**9.4 ± 2**	**10.2 ± 2.1**	**8.5 ± 1.4**	**<0.001**
Platelets count (G/L)	**115 ± 73**	**126 ± 73**	**102 ± 71**	**0.002**
PT (%)	**55.3 ± 15.5**	**63.3 ± 12.6**	**45.5 ± 13.0**	**<0.001**
INR	**1.6 ± 0.4**	**1.4 ± 0.2**	**1.9 ± 0.5**	**<0.001**
Fibrinogen (g/L)	**2.8 ± 1.1**	**3.2 ± 1.2**	**2.1 ± 0.7**	**<0.001**
Lactate (mmol/L)	1.8 ± 1.3	1.6 ± 0.5	2.1 ± 1.8	0.213
Serum sodium (mmol/L)	135 ± 5	135 ± 5	136 ± 5	0.094
Albumin (g/L)	**29 ± 6**	**30 ± 5**	**27 ± 5**	**<0.001**
AST (UI/L)	**84 ± 42**	**48 ± 35**	**127 ± 130**	**<0.001**
ALT (UI/L)	**47 ± 65**	**29 ± 23**	**69 ± 89**	**<0.001**
Bilirubin (μmol/L)	**55 ± 74**	**22 ± 16**	**100 ± 96**	**<0.001**
Ammonemia (μmol/L)	**64 ± 42**	**54 ± 36**	**76 ± 46**	**<0.001**
NT_proBNP (ng/ml)	520 ± 223	577 ± 286	439 ± 689	0.768
CLIF_C_AD	50 ± 8	49 ± 6	52 ± 10	0.161
Ascites at admission, n (%)	**135 (71)**	**84 (80.0%)**	**51 (60.71%)**	**0.006**
OHE at admission, n (%)	**31 (16)**	**0 (0)**	**31 (36.5)**	**<0.001**
PPG	**7 (6-9)**	**7 (6-9)**	**7 (6-9)**	**0.145**
Treatment with lactulose, n (%)	**97 (50.7)**	**36 (34.3)**	**61 (71.8)**	**<0.001**
Treatment with rifaximin, n (%)	**64 (33.5)**	**54 (50.9)**	**10 (11.8)**	**<0.001**

Bold corresponds to statisitically different characterstics. Values are expressed as mean, median or absolute value (%). Student's *t* test was used for group comparisons of normally distributed continuous variables. Group comparisons of categorical variables were performed using Chi-squared test. A *p* value <0.05 was considered significant. ALD, alcohol-related liver disease; ALT, alanine aminotransferase; AST, aspartate aminotransferase; AVB, acute variceal bleeding; CLIF-C-AD, chronic liver failure-consortium- acute decompensation; HCC, hepatocellular carcinoma; INR, international normalized ratio; L3SMI, skeletal muscle index; MASLD, metabolic-dysfunction associated steatotic liver disease; MELD, model for end-stage liver disease; MetALD, metabolic and alcohol-related liver disease; NT-proBNP, N-terminal pro B-type natriuretic peptide; OHE, overt hepatic encephalopathy; PPG, portal pressure gradient; PT, prothrombin time ratio.
*Data available in 190 patients.

remained associated with the development of OHE were previous cardiac disease (HR 4.12, 95% CI 1.21-14.1, *p* = 0.02) and sarcopenia (HR 3.56, 95% CI 1.27-9.96, *p* = 0.016). Cumulative incidence of OHE after pTIPS in patients with or without ammonia >ULN is shown in Fig. S2 (*p* = 0.51).

Development of OHE during follow-up in patients treated with elective TIPS

Study cohort

The 1-year cumulative incidence of OHE considering death and LT as competing risks after elective TIPS was 38% (95% CI 29-48), and was not statistically different from that of the pTIPS group (*p* = 0.6, Fig. 1). Baseline characteristics of patients treated with elective TIPS according to the development of OHE are given in Table S4. The mean delay between elective TIPS placement and OHE was 19 [9-113] days. In the elective TIPS group, among the 41 patients who developed OHE, HE resolution occurred in 32 patients (78%). Fig. 3 shows the evolution of OHE and patient prognosis according to resolution of OHE after elective TIPS.

In univariate analysis, previous cardiac disease (HR 2.61, 95% CI 1.41-4.82, *p* = 0.001), and baseline ammonia (HR 1.01, 95% CI 1.01-1.02, *p* <0.001) and albumin (HR 0.90, 95% CI 0.85-0.96, *p* <0.001) levels were significantly associated with

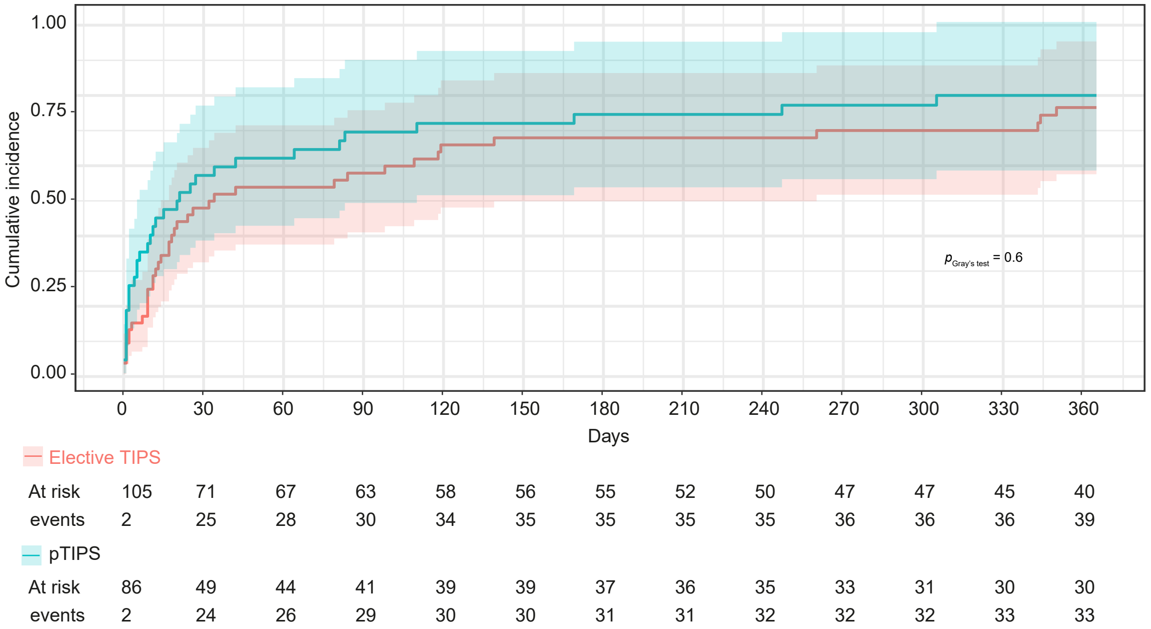

Fig. 1. One-year cumulative incidence of OHE in pTIPS and elective TIPS groups, with LT and death as competing risks. The cumulative incidence of OHE was not different in the pTIPS and in the elective TIPS groups (p = 0.40). The Fine and Gray model was used and comparison between pTIPS and elective TIPS patients was performed using Gray's test. LT, liver transplantation; OHE, overt hepatic encephalopathy; (p)TIPS, (preemptive) transjugular intrahepatic portosystemic shunt.

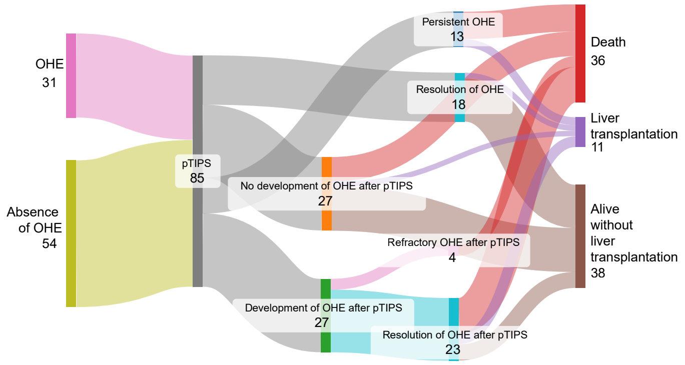

Fig. 2. Evolution of patients with OHE at admission after pTIPS placement. Sankay diagram illustrating the outcomes after pTIPS according to OHE at admission and after pTIPS. OHE, overt hepatic encephalopathy; (p)TIPS, (preemptive) transjugular intrahepatic portosystemic shunt.

post-TIPS OHE occurrence, whereas patients treated with rifaximin (HR 0.41, 95% CI 0.21-0.79, p = 0.008) experienced significantly less OHE episodes after TIPS. In multivariate analysis, a previous cardiac disease (HR 2.39 95% CI 1.25-4.59, p = 0.009), and baseline serum ammonia (HR 1.01, 95% CI 1.00-1.02, p = 0.022) and baseline albumin (HR 0.93, 95% CI 0.88-0.99, p = 0.018) levels were associated with OHE in the elective group (Table 3). Post-TIPS PPG and a previous episode of OHE were not significantly associated with post-TIPS OHE. Cumulative incidence of OHE after elective TIPS in patients with or without baseline ammonia >ULN is shown in Fig. S3 (p = 0.04).

Validation cohort

In the validation cohort, post-TIPS PPG and a previous episode of OHE were similarly not significantly associated with post-TIPS OHE. The only factor associated with post-TIPS OHE in multivariate analysis was serum creatinine (Table S5).

Mortality and liver transplantation

During follow-up, 71/191 patients (37.2%) died and 29/191 (15.2%) were transplanted, without any difference between pTIPS and elective TIPS groups (41.8% *vs.* 33.3%, p = 0.29 for mortality, and 12.8% *vs.* 17.1%, p = 0.53 for LT).

Univariate and multivariate analyses of factors associated with death after pTIPS, considering LT as a competing event, are shown in Table 4. In univariate analysis, factors associated with death were age (HR 1.04, 95% CI 1.01-1.07, p = 0.023), bilirubin (HR 1.00, 95% CI 1.00-1.01, p = 0.019), creatinine (HR 1.01, 95% CI 1.00-1.01, p = 0.042), MELD score (HR 1.07, 95% CI 1.01-1.13, p = 0.018), Child-Pugh score (HR 1.46, 95% CI 1.15-1.87, p = 0.002), and persistent OHE after pTIPS (HR

Table 2. Univariate and multivariate analysis of baseline risk factors for post-TIPS OHE (LT competing risk) in the pTIPS group (n=39).

	Univariate analysis for OHE in pTIPS group sHR [95% CI], *p* value	*p* value	Multivariate analysis for OHE in pTIPS group sHR [95% CI], *p* value	*p* value
Age	1.01 [0.98-1.05]	0.32		
Type 2 diabetes	2.18 [1.06-4.50]	0.03	0.67 [0.15-2.93]	0.6
Previous OHE	1.56 [0.76-3.22]	0.23		
Previous cardiac disease	**2.16 [1.03-4.50]**	**0.04**	**4.12 [1.21-14.1]**	**0.02**
Sarcopenia	**2.91 [1.03-8.27]**	**0.04**	**3.56 [1.27-9.96]**	**0.016**
Baseline ammonia	1.00 [0.99-1.01]	0.74		
Platelets count	1.00 [1.00-1.00]	0.60		
Serum sodium	1.00 [0.93-1.08]	0.96		
Albumin	0.97 [0.91-1.03]	0.34		
Creatinine	1.00 [1.00-1.01]	0.11		
MELD score	0.98 [0.93- 1.02]	0.31		
Treatment with lactulose	1.08 [0.55-2.12]	0.82		
Large spontaneous shunts	1.76 [0.89-3.47]	0.06	1.72 [0.83-3.56]	0.14
PPG	0.95 [0.81-1.11]	0.52		

Bolded values indicate statistically significant differences (*p* <0.05) (Fine and Gray models). MELD, model for end-stage liver disease; OHE, overt hepatic encephalopathy; PPG, portal pressure gradient; sHR, subdistribution hazard ratio.

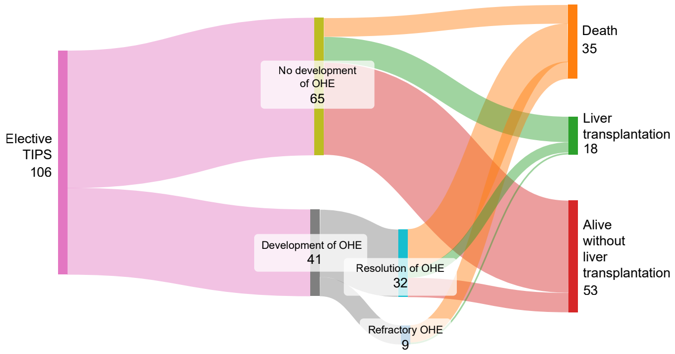

Fig. 3. Evolution of patients with or without OHE after elective TIPS placement. Sankay diagram illustrating the outcomes after elective TIPS according to the development of OHE after TIPS. OHE, overt hepatic encephalopathy; TIPS, transjugular intrahepatic portosystemic shunt.

7.63, 95% CI 3.18-18.1, *p* <0.001) but not OHE at admission. In multivariate analysis, the factors that remained independently associated with mortality were age (HR 1.08, 95% CI 1.02-1.13, *p* = 0.006), MELD score (HR 1.10, 95% CI 1.01-1.29, *p* = 0.04) and persistent OHE after pTIPS (HR 9.25, 95% CI 3.46-25.8, *p* <0.001).

In the landmark analysis, we evaluated the prognostic value of OHE at 1, 3 and 6 months after pTIPS placement. We found that OHE was significantly associated with death irrespective of time frame (Fig. S4, *p* <0.05 for all analyses).

In the elective TIPS group, univariate analysis showed that age (HR 1.07, 95% CI 1.02-1.12, *p* = 0.01), a previous cardiac disease (HR 2.88, 95% CI 1.40-5.92, *p* = 0.004), creatinine (HR 1.01, 95% CI 1.00-1.02, *p* <0.001), MELD score (HR 1.09, 95% CI 1.00-1.18, *p* = 0.048), the development of OHE after TIPS (HR 3.40, 95% CI 1.57-7.36, *p* = 0.002), and refractory OHE (HR 5.14, 95% CI 2.42-10.9, *p* <0.001), were associated with death. In multivariate analysis, age (HR 1.06, 95% CI 1.01-1.12, *p* = 0.03), MELD score (HR 1.10, 95% CI 1.01-1.29, *p* = 0.04), OHE after TIPS (HR 2.94, 95% CI 1.21-7.11, *p* = 0.017) and refractory OHE after TIPS (HR 4.92, 95% CI 2.25-10.8, *p* <0.001) remained independently associated with death (Table 5). Fig. 4 shows transplant-free survival in patients treated with pTIPS according to persistent OHE after pTIPS.

Table 3. Univariate and multivariate analysis of baseline risk factors for OHE (death and LT competing risk) in the elective TIPS group (n = 41).

	Univariate analysis for OHE in pTIPS group sHR [95% CI], *p* value	*p* value	Multivariate analysis for OHE in pTIPS group sHR [95% CI], *p* value	*p* value
Age	1.01 [0.97-1.05]	0.52		
Type 2 diabetes	1.74 [0.97-3.15]	0.06		
Previous OHE	0.63 [0.28-1.40]	0.27		
Previous cardiac disease	**2.61 [1.41-4.82]**	**0.001**	**2.39 [1.25-4.59]**	**0.009**
Baseline ammonia	**1.01 [1.01-1.02]**	**<0.001**	**1.01 [1.00-1.02]**	**0.022**
Sarcopenia	0.82 [0.35-1.91]	0.65		
Platelets count	1.00 [1.00-1.00]	0.28		
Serum sodium	1.02 [0.96-1.08]	0.56		
Albumin	**0.90 [0.85-0.96]**	**<0.001**	**0.93 [0.88-0.99]**	**0.018**
Creatinine	1.00 [1.00-1.01]	0.66		
MELD score	1.02 [0.95-1.10]	0.52		
Treatment with rifaximin	0.41 [0.21-0.79]	0.008	0.62 [0.30-1.27]	0.20
Treatment with lactulose	0.97 [0.49-1.90]	0.92		
Large spontaneous shunts	1.54 [0.79-3.02]	0.21		
PPG	1.01 [0.90-1.13]	0.87		

Bolded values indicate statistically significant differences (*p* <0.05) (Fine and Gray models). MELD, model for end-stage liver disease; OHE, overt hepatic encephalopathy; PPG, portal pressure gradient; sHR, subdistribution hazard ratio.

Table 4. Analysis of baseline risk factors for death (LT competing risk) in the pTIPS group.

	Univariate analysis for death or LT in pTIPS group sHR [95% CI], *p* value	*p* value
Age	**1.04 [1.01-1.07]**	**0.023**
Type 2 diabetes	0.85 [0.35-2.05]	0.72
Previous cardiac disease	1.10 [0.32-3.85]	0.88
OHE at admission	0.72 [0.37-1.41]	0.34
Bilirubin	**1.00 [1.00-1.01]**	**0.019**
Platelets count	1.00 [1.00-1.00]	0.65
Albumin	0.98 [0.92-1.05]	0.56
Baseline ammonia	1.00 [0.99-1.01]	0.79
Creatinine	**1.01 [1.00-1.02]**	**0.042**
MELD score	**1.07 [1.01-1.13]**	**0.018**
Child-Pugh score	**1.46 [1.15-1.87]**	**0.002**
HVPG	1.00 [0.89-1.12]	0.99
PPG	0.95 [0.81-1.11]	0.99
OHE after pTIPS	2.16 [0.98-4.73]	0.056
Persistent OHE after pTIPS	**7.63 [3.18-18.1]**	**<0.001**
**Model 1**	**Multivariate analysis for death or LT in pTIPS group sHR [95% CI], *p* value**	***p* value**
Age	1.08 [1.02-1.13]	0.006
MELD score	1.10 [1.01-1.29]	0.04
OHE after TIPS	2.08 [0.93-4.70]	0.07
**Model 2**		
Age	**1.05 [1.02-1.09]**	**0.003**
Bilirubin	1.00 [1.00-1.01]	0.12
Persistent OHE after TIPS	**9.25 [3.46-25.8]**	**<0.001**

Bolded values indicate statistically significant differences (*p* <0.05) (Fine and Gray models). HVPG, hepatic venous pressure gradient; MELD, model for end-stage liver disease; OHE, overt hepatic encephalopathy; PPG, portal pressure gradient; sHR, subdistribution hazard ratio; TIPS, transjugular intrahepatic portosystemic shunt.

Table 5. Analysis of baseline risk factors for death (LT competing risk) in the elective TIPS group.

	Univariate analysis for death or LT in pTIPS group sHR [95% CI], *p* value	*p* value
Age	**1.07 [1.02-1.12]**	**0.01**
Type 2 diabetes	1.50 [0.80-2.78]	0.20
Previous cardiac disease	**2.88 [1.40-5.92]**	**0.004**
Bilirubin	1.01 [1.00-1.03]	0.17
Platelets count	1.00 [1.00-1.00]	0.28
Albumin	0.97 [0.85-1.06]	0.56
Baseline ammonia	1.01[1.00-1.01]	0.18
Creatinine	**1.01 [1.00-1.01]**	**<0.001**
MELD score	**1.09 [1.00-1.18]**	**0.048**
Child-Pugh score	1.08 [0.87-1.33]	0.48
HVPG	1.04 [0.94-1.14]	0.47
PPG	1.01 [0.90-1.13]	0.87
OHE after TIPS	**3.40 [1.57-7.36]**	**0.002**
Refractory OHE after TIPS	**5.14 [2.42-10.90]**	**<0.001**
**Model 1**	**Multivariate analysis for death or LT in pTIPS group sHR [95% CI], *p* value**	***p* value**
Age	**1.06 [1.01-1.12]**	**0.03**
MELD	**1.10 [1.01-1.29]**	**0.04**
OHE after TIPS	**2.94 [1.21-7.11]**	**0.017**
**Model 2**		
Age	**1.05 [0.99-1.11]**	**0.08**
MELD	**1.11 [1.02-1.24]**	**0.02**
Refractory OHE after TIPS	**4.92 [2.25-10.8]**	**<0.001**

Bolded values indicate statistically significant differences (*p* <0.05) (Fine and Gray models). HVPG, hepatic venous pressure gradient; MELD, model for end-stage liver disease; OHE, overt hepatic encephalopathy; PPG, portal pressure gradient; sHR, subdistribution hazard ratio; TIPS, transjugular intrahepatic portosystemic shunt.

In the landmark analysis, we evaluated the prognostic value of OHE at 1, 3 and 6 months after elective TIPS placement. We found that OHE was significantly associated with death irrespective of time frame (Fig. S4, *p* <0.05 for all analyses).

Sensitivity analysis in the subgroups of patients treated with elective TIPS either for hydrothorax/ascites or other causes

Outcomes (OHE, death or LT) were not statistically different in patients treated with elective TIPS, either for hydrothorax/ascites or other causes (Table S6).

Discussion

In this retrospective study of patients with cirrhosis from a prospective TIPS cohort, we aimed to evaluate the occurrence of OHE after pTIPS, its impact on long-term outcomes, and to compare it with that of patients who underwent elective TIPS. Our main findings were:[1] risk factors for OHE differed according to TIPS indication;[2] OHE was independently associated with poorer survival after elective TIPS but not after pTIPS; and[3] persistent OHE was independently associated with worse survival after pTIPS.

OHE occurrence after TIPS is probably the main reason physicians may be reluctant to perform TIPS,[26] due to both the severe prognosis of OHE[10] and the potentially persistent cognitive impairment,[28] leading to reduced quality of life and a substantial burden on patients and society. Although increasing evidence supports improved survival with pTIPS,[1,25] its reported use remains low (<10%) even when indicated.[27,29]

The current study is the first one, to our knowledge, that aimed to evaluate risk factors for OHE after pTIPS, as well as the prognostic value of OHE after TIPS on mortality in both elective and preemptive indications. Indeed, in a previous observational study,[4] we found that pTIPS conferred a survival benefit despite the presence of OHE at admission, when compared to classical secondary prophylaxis including non-selective beta blockers and repeated band ligation. Moreover, pTIPS was not associated with a higher rate of further OHE. Nevertheless, we could not analyze factors associated with a higher risk of OHE after pTIPS. Here, previous cardiac disease and sarcopenia were independent factors associated with OHE after pTIPS. While sarcopenia is a well-known risk factor for OHE after elective TIPS, previous cardiac disease has, to our knowledge, not been reported before. Indeed, other classical risk factors for OHE after elective TIPS include older age, poor liver function, previous OHE, hyponatremia, renal failure, and minimal HE.[8,9,17–19] None of these classical factors were found in our study, even in elective situations. This may be explained by differences in baseline characteristics: our patients were younger, and over 50% received rifaximin as primary prophylaxis, an approach that could modify the natural history of OHE after TIPS. Moreover, we probably modified our clinical practices after the clear identification of risk factors for OHE, such as PPG. We avoid lowering PPG <8 mmHg after elective TIPS, an approach that may explain why PPG is not consistently associated with OHE after TIPS. Similar findings have been reported elsewhere; for example, a large Italian

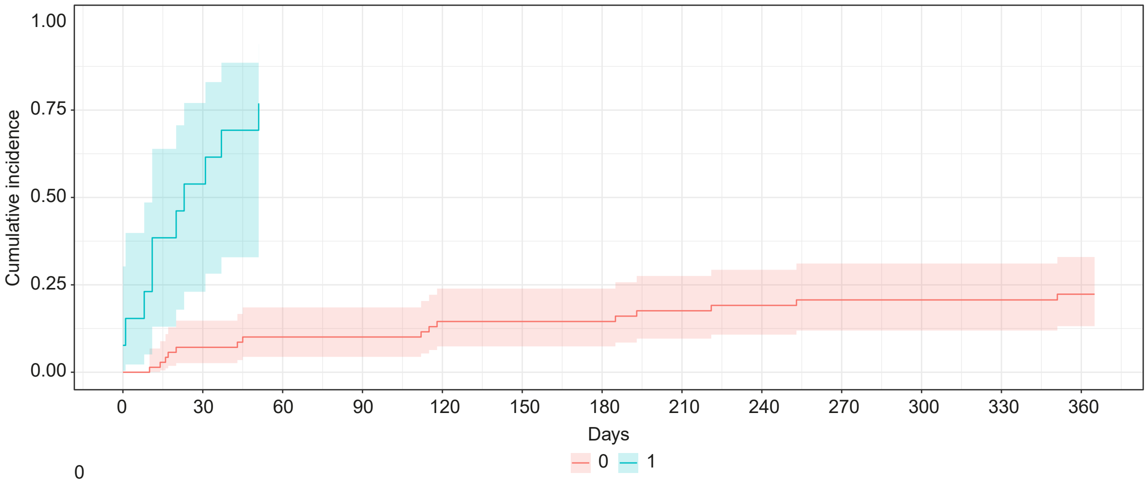

0													
At risk	73	63	57	57	54	54	53	49	47	46	44	44	43
events	0	5	7	7	10	10	10	12	13	14	14	14	15
1													
At risk	13	3	0	0	0	0	0	0	0	0	0	0	0
events	1	7	10	10	10	10	10	10	10	10	10	10	10

Fig. 4. Cumulative incidence of death in pTIPS group in patients with or without OHE after pTIPS. The cumulative incidence of death was significantly higher in patients with persistent OHE after pTIPS (*p* <0.001). The Fine and Gray model was used and comparison between patients with or without persistent OHE was performed using Gray's test. OHE, overt hepatic encephalopathy; (p)TIPS, (preemptive) transjugular intrahepatic portosystemic shunt.

study including 614 patients and a multicenter German study[30] both found no association between PPG and OHE risk. In our series, rifaximin was not protective against OHE, likely due to the small number of treated patients (n = 54 in the elective TIPS group), which limited statistical power. Similar limitations were noted in several other underpowered studies.[31–33] By contrast, the positive RCT by Bureau *et al.*,[20] which included 197 patients (97 treated with rifaximin), demonstrated a protective effect.

Considering only the standard contraindications for TIPS, it is important to remember that elective TIPS for ascites improves survival[7] and reduces the risk of further decompensation in patients receiving secondary prophylaxis for variceal bleeding.[1] The epidemiology of cirrhosis has changed,[34] with a higher proportion of patients with MASLD and MetALD now hospitalized for decompensation, as in this study. Consequently, a significant number of patients had pre-existing cardiac disease of various etiologies (ischemic or arrhythmic), which was associated with the subsequent development of OHE after both pTIPS and elective TIPS. This novel finding may be related to concomitant cerebrovascular disease, which can impair neurocognitive reserve and facilitate OHE.

Baseline ammonia was also associated with a higher risk of OHE after elective TIPS. Although elevated ammonia is recognized as part of OHE pathogenesis, its predictive value before TIPS has not previously been described. Recent data suggest that ammonia may not only help rule out differential diagnoses of OHE but, when elevated, can also predict mortality in patients hospitalized for acute-on-chronic liver failure or OHE development in clinically stable outpatients.[35,36] These findings highlight the need to consider new risk factors for OHE, which require further validation.

Another important finding of the current study is the prognostic value of persistent OHE after pTIPS, as well as liver function, as assessed by a higher MELD score. To our knowledge, this result has never been described. Until now, the prognostic value of OHE has been documented in AVB but not well described in the pTIPS era. Indeed, OHE was previously identified as a predictor of poor prognosis in high-risk patients with AVB,[4,12] with a prevalence as high as 40% at admission.[4] Nevertheless, it was difficult to describe the exact clinical course of OHE at admission in this later study. Herein, we had the opportunity to clearly identify that patients with OHE at admission who did not recover after pTIPS had the poorest prognosis, as all of them died or were transplanted. Importantly, the occurrence of episodic OHE after pTIPS was not associated with death or LT in multivariate analysis. First, they support systematic discussion of TIPS and LT even in emergency settings, since transplantation may be the only therapeutic option for patients with persistent OHE after pTIPS. Second, management of OHE in the context of AVB should not be overlooked, and the role of rifaximin as a prophylactic treatment warrants rapid evaluation. Finally, pTIPS may serve as a bridge to LT even in patients at high risk of OHE.

Another strength of our study was the possibility to compare the prognosis of OHE in two different situations, elective and preemptive, in consecutive patients treated with TIPS. In this context, the recent study by Nardelli suggested that post-elective TIPS OHE was not associated with poorer survival. Our findings are a little different, because in our series post-TIPS OHE occurrence was associated with worse survival. Notably, occurrence of OHE was rather fast, with a median delay of less than 2 months, whereas this delay was higher in Nardelli *et al.*'s paper.[8] We would like to emphasize

that our findings in elective situations are completely in line with previous results showing that "early" OHE after TIPS, *i.e.* occurring within the 3 months after TIPS or even within the first month[14] is associated with higher mortality. Last, refractory OHE after elective TIPS is associated with death or LT, which seems quite logical. Once again, these data should encourage the systematic discussion of LT together with TIPS, as LT is currently the only curative treatment that improves survival in end-stage liver disease.

We must acknowledge that our study has several limitations. It is limited by its small sample size and single-center design, which may introduce bias related to local clinical practice. For example, only two patients in our elective TIPS cohort underwent underdilation, a technique previously associated with lower OHE incidence;[35] although statistical analysis was not possible, neither developed OHE. Systematic embolization of shunts was not performed, despite some studies suggesting this approach may reduce OHE.[37,38] Nevertheless, likely due to changes in patient management over time, we did not observe higher OHE rates than typically reported. Besides this, the "classical" risk factors of OHE after elective TIPS, such as age, chronic kidney failure, low PPG, and previous OHE, were not confirmed in the current study. This may reflect changes in patient selection for TIPS over the last decade, as we systematically discussed TIPS and LT together, considering TIPS as a potential bridge to LT. Therefore, we treated a higher rate of patients with a previous episode of OHE than in other studies (21% compared to 12% in the RCT from Bureau *et al.*), as salvage LT could be considered in most of our patients, in case of occurrence of refractory OHE after TIPS.[26] Moreover, we considered that the control of OHE before TIPS may have an influence on OHE after TIPS,[39] as data on the management of the previous OHE were lacking in older studies[9]. Additionally, with the renewed interest in ammonia, we systematically evaluated ammonia before TIPS, which is not available in most existing cohorts. Finally, the epidemiology of cirrhosis has shifted, with a growing prevalence of steatotic-related disease (MASLD and MetALD accounted for 40% of cases in our cohort, compared with >80% alcohol-related cirrhosis in other cohorts [[20],[40]]). This change may be clinically relevant, as brain reserve could differ between patients with MASLD and those with ALD, potentially altering the natural history of OHE after TIPS.[41]

In conclusion, OHE incidence was similar after pTIPS and elective TIPS in our recent series. However, the associated risk factors differed between these settings. Regardless of TIPS indication, persistent or refractory OHE after the procedure is associated with poor prognosis, and prompt consideration of liver transplantation is warranted.

Affiliations

[1]*AP-HP, Sorbonne Université, Liver Intensive Care Unit, Hepatogastroenterology Department, La Pitié-Salpêtrière Hospital, 47-83 Boulevard de l'Hôpital, Paris 75013, France;* [2]*INSERM UMR_S 938, Centre de recherche Saint-Antoine, Maladies métaboliques, biliaires et fibro-inflammatoire du foie, Institute of Cardiometabolism and Nutrition (ICAN), Paris, France;* [3]*Brain-Liver Pitié-Salpêtrière Study group (BLIPS), France;* [4]*Radiology department, La Pitié-Salpêtrière Hospital, 47-83 Boulevard de l'Hôpital, Paris 75013, France;* [5]*AP-HP. Sorbonne Université, Neurology Intensive Care Unit, Neurology Department, La Pitié-Salpêtrière Hospital, 47-83 Boulevard de l'Hôpital, Paris 75013, France;* [6]*OncoRay-National Center for Radiation Research in Oncology, Faculty of Medicine and University Hospital Carl Gustav Carus, Technische Universität Dresden, Helmholtz-Zentrum Dresden-Rossendorf, Fetscherstraße 74, 01307, Dresden, Germany;* [7]*University Hospital of Toulouse, Toulouse, France;* [8]*Department of Hepato-gastroenterology, Hepatology and Liver Transplantation Unit, Saint Eloi Hospital, Univ Montpellier, Montpellier, France*

Abbreviations

ALD, alcohol-related liver disease; AVB, acute variceal bleeding; HCC, hepatocellular carcinoma; HE, hepatic encephalopathy; LT, liver transplantation; MASLD, metabolic-dysfunction associated steatotic liver disease; MELD, model for end-stage liver disease; MetALD, metabolic and alcohol-related liver disease; OHE, overt hepatic encephalopathy; PPG, portal pressure gradient; (p)TIPS, (preemptive) transjugular intrahepatic portosystemic shunt.

Financial support

The authors did not receive any financial support to produce this manuscript.

Conflict of interest

MR: Speaker for Gore, Abbvie. CB: None. CR: Speaker for Gore. PS: None. PP: None. MJ: None. LK: None. NW: Consultant for Alexion, Okwin. ARM: None. BJ: None. SM: None. LR: None. CB: Speaker for Gore. JUB: None. DT: Speaker for Gore, Gilead, Abbvie, consulting Alfasigma.

Please refer to the accompanying ICMJE disclosure forms for further details.

Authors' contribution

MR: study conception, drafting the manuscript, critical review of the manuscript. CB: data collection, critical review of the manuscript. CR: management of patients, critical review of the manuscript. PP: data collection, critical review of the manuscript. MJ: data collection, critical review of the manuscript. PS: critical review of the manuscript, statistical analysis. ARM: data collection. BJ: data collection. LK: critical review of the manuscript. NW: critical review of the manuscript. SM: data collection, critical review of the manuscript. DT: study conception, drafting the manuscript, critical review of the manuscript.

Data availability

Data available on reasonable request.

Supplementary data

Supplementary data to this article can be found online at https://doi.org/10.1016/j.jhepr.2025.101548.

References

[1] Larrue H, D'Amico G, Olivas P, et al. TIPS prevents further decompensation and improves survival in patients with cirrhosis and portal hypertension in an individual patient data meta-analysis. J Hepatol 2023 Sep;79(3):692–703.

[2] Horhat A, Bureau C, Thabut D, et al. Transjugular intrahepatic portosystemic shunt in patients with cirrhosis: indications and posttransjugular intrahepatic portosystemic shunt complications in 2020. United Eur Gastroenterol J 2021 Mar;9(2):203–208.

[3] Larrue H, Allaire M, Weil-Verhoeven D, et al. Groupe collaboratif AFEF. French guidelines on TIPS: indications and modalities. Liver Int 2024 Sep;44(9):2125–2143.

[4] Rudler M, Hernández-Gea V, Procopet BD, et al. For International Variceal Bleeding Observational Study Group: a Baveno Cooperation. Hepatic encephalopathy is not a contraindication to pre-emptive TIPS in high-risk patients with cirrhosis with variceal bleeding. Gut 2023 Apr;72(4):749–758.

[5] Bouzbib C, Cluzel P, Sultanik P, et al. Prognosis of patients undergoing salvage TIPS is still poor in the preemptive TIPS era. Clin Res Hepatol Gastroenterol 2021 Nov;45(6):101593.

[6] Walter A, Rudler M, Olivas P, et al., Salvage TIPS Group. Combination of model for end-stage liver disease and lactate predicts death in patients treated with salvage transjugular intrahepatic portosystemic shunt for refractory variceal bleeding. Hepatology 2021 Oct;74(4):2085–2101.

[7] Bureau C, Thabut D, Oberti F, et al. Transjugular intrahepatic portosystemic shunts with covered stents increase transplant-free survival of patients with cirrhosis and recurrent ascites. Gastroenterology 2017 Jan;152(1):157–163. https://doi.org/10.1053/j.gastro.2016.09.016. Epub 2016 Sep 20. Erratum in: Gastroenterology. 2017 Sep;153(3):870.

[8] Nardelli S, Riggio O, Marra F, et al. Episodic overt hepatic encephalopathy after transjugular intrahepatic portosystemic shunt does not increase mortality in patients with cirrhosis. J Hepatol 2024 Apr;80(4):596–602.

[9] Bai M, Qi X, Yang Z, et al. Predictors of hepatic encephalopathy after transjugular intrahepatic portosystemic shunt in cirrhotic patients: a systematic review. J Gastroenterol Hepatol 2011 Jun;26(6):943–951. https://doi.org/10.1111/j.1440-1746.2011.06663.x. PMID: 21251067.

[10] Jepsen P, Ott P, Andersen PK, Sørensen HT, et al. Hepatology 2010 May;51(5):1675–1682.

[11] Riggio O, Celsa C, Calvaruso V, et al. Hepatic encephalopathy increases the risk for mortality and hospital readmission in decompensated cirrhotic patients: a prospective multicenter study. Front Med (Lausanne) 2023 May 25;10:1184860.

[12] Rudler M, Bureau C, Carbonell N, et al., French Club for the Study of Portal Hypertension (CFEHTP). Recalibrated MELD and hepatic encephalopathy are prognostic factors in cirrhotic patients with acute variceal bleeding. Liver Int 2018 Mar;38(3):469–476.

[13] Cordoba J, Ventura-Cots M, Simón-Talero M, et al. CANONIC Study Investigators of EASL-CLIF Consortium. Characteristics, risk factors, and mortality of cirrhotic patients hospitalized for hepatic encephalopathy with and without acute-on-chronic liver failure (ACLF). J Hepatol 2014 Feb;60(2):275–281. https://doi.org/10.1016/j.jhep.2013.10.004. Epub 2013 Oct 12. PMID: 24128414.

[14] Hartl L, Kornfehl A, Reiberger T. Does encephalopathy with early onset after TIPS impact on mortality? J Hepatol 2024 Dec;81(6):e269–e270.

[15] Rudler M, Weiss N, Perlbarg V, et al. Combined diffusion tensor imaging and magnetic resonance spectroscopy to predict neurological outcome before transjugular intrahepatic portosystemic shunt. Aliment Pharmacol Ther 2018 Oct;48(8):863–874.

[16] Salerno F, Cammà C, Enea M, et al. Transjugular intrahepatic portosystemic shunt for refractory ascites: a meta-analysis of individual patient data. Gastroenterology 2007 Sep;133(3):825–834.

[17] Nardelli S, Gioia S, Pasquale C, et al. Cognitive impairment predicts the occurrence of hepatic encephalopathy after transjugular intrahepatic portosystemic shunt. Am J Gastroenterol 2016 Apr;111(4):523–528.

[18] Nardelli S, Lattanzi B, Torrisi S, et al. Sarcopenia is risk factor for development of hepatic encephalopathy after transjugular intrahepatic portosystemic shunt placement. Clin Gastroenterol Hepatol 2017 Jun;15(6):934–936.

[19] Berlioux P, Robic MA, Poirson H, et al. Pre-transjugular intrahepatic portosystemic shunts (TIPS) prediction of post-TIPS overt hepatic encephalopathy: the critical flicker frequency is more accurate than psychometric tests. Hepatology 2014 Feb;59(2):622–629.

[20] Bureau C, Thabut D, Jezequel C, et al. The use of rifaximin in the prevention of overt hepatic encephalopathy after transjugular intrahepatic portosystemic shunt : a randomized controlled trial. Ann Intern Med 2021 May;174(5):633–640.

[21] European Association for the Study of the Liver. EASL Clinical Practice Guidelines on the management of hepatic encephalopathy. J Hepatol 2022 Sep;77(3):807–824. https://doi.org/10.1016/j.jhep.2022.06.001. Epub 2022 Jun 17. Erratum in: J Hepatol. 2023 Nov;79(5):1340.

[22] Vidal-González J, Martínez J, Mulay A, et al. Baveno VI-SPSS group from the Baveno Cooperation. Evolution of spontaneous portosystemic shunts over time and following aetiological intervention in patients with cirrhosis. JHEP Rep 2023 Nov 30;6(2):100977.

[23] de Franchis R, Bosch J, Garcia-Tsao G, et al. Baveno VII - renewing consensus in portal hypertension. J Hepatol 2022 Apr;76(4):959–974. https://doi.org/10.1016/j.jhep.2021.12.022. Epub 2021 Dec 30. Erratum in: J Hepatol. 2022 Jul;77(1):271.

[24] de Franchis R, VI Faculty Baveno. Expanding consensus in portal hypertension: report of the Baveno VI Consensus Workshop: stratifying risk and individualizing care for portal hypertension. J Hepatol 2015 Sep;63(3):743–752.

[25] Nicoară-Farcău O, Han G, Rudler M, et al. pre-emptive TIPS individual data metanalysis, International Variceal Bleeding Study and Baveno Cooperation Study groups. Pre-emptive TIPS in high-risk acute variceal bleeding. An updated and revised individual patient data meta-analysis. Hepatology 2024 Mar 1;79(3):624–635.

[26] Rudler M, Savier E, Alioua I, et al. TIPS and liver transplantation should always be discussed together. J Hepatol 2021 Oct;75(4):1000–1001.

[27] Thabut D, Pauwels A, Carbonell N, et al. Cirrhotic patients with portal hypertension-related bleeding and an indication for early-TIPS: a large multicentre audit with real-life results. J Hepatol 2017 Dec 14;68(1):73–81.

[28] Bajaj JS, Schubert CM, Heuman DM, et al. Persistence of cognitive impairment after resolution of overt hepatic encephalopathy. Gastroenterology 2010 Jun;138(7):2332–2340.

[29] Hernández-Gea V, Procopet B, Giráldez Á, et al. International variceal bleeding observational study group and Baveno cooperation. Preemptive-TIPS improves outcome in high-risk variceal bleeding: an observational study. Hepatology 2019 Jan;69(1):282–293.

[30] Kabelitz MA, Sandmann L, Praktiknjo M, et al. German cirrhosis study group. Early occurrence of hepatic encephalopathy following transjugular intrahepatic portosystemic shunt insertion is linked to impaired survival: a multicenter cohort study. Clin Gastroenterol Hepatol 2025 Mar;15. S1542-3565(25)00203-4.

[31] Riggio O, Masini A, Efrati C, et al. Pharmacological prophylaxis of hepatic encephalopathy after transjugular intrahepatic portosystemic shunt: a randomized controlled study. J Hepatol 2005 May;42(5):674–679.

[32] Seifert LL, Schindler P, Schoster M, et al. Recurrence of hepatic encephalopathy after TIPS: effective prophylaxis with combination of lactulose and rifaximin. J Clin Med 2021 Oct 17;10(20):4763.

[33] Labenz C, Schmidtke L, Pitton MB, et al. Ammonia levels after TIPS insertion identify patients at high risk of hepatic encephalopathy. Clin Gastroenterol Hepatol 2025 May;10. S1542-3565(25)00367-2.

[34] Shalimar Sheikh MF, Mookerjee RP, et al. Prognostic role of ammonia in patients with cirrhosis. Hepatology 2019 Sep;70(3):982–994.

[35] Tranah TH, Ballester MP, Carbonell-Asins JA, et al. Plasma ammonia levels predict hospitalisation with liver-related complications and mortality in clinically stable outpatients with cirrhosis. J Hepatol 2022 Dec;77(6):1554–1563.

[36] Schepis F, Vizzutti F, Garcia-Tsao G, et al. Under-dilated TIPS associate with efficacy and reduced encephalopathy in a prospective, non-randomized study of patients with cirrhosis. Clin Gastroenterol Hepatol 2018 Jul;16(7):1153–1162.e7.

[37] Lv Y, Chen H, Luo B, et al. Concurrent large spontaneous portosystemic shunt embolization for the prevention of overt hepatic encephalopathy after TIPS: a randomized controlled trial. Hepatology 2022 Sep;76(3):676–688.

[38] Wang Z, Xu XY, Liu CY, et al. Evaluation of the efficacy and safety of transjugular intrahepatic portosystemic shunt combined with concurrent antegrade embolization of large spontaneous portosystemic shunts. J Med Imaging Radiat Oncol 2025 Mar;69(2):228–236.

[39] Saab S, Zhao M, Asokan I, et al. History of hepatic encephalopathy is not a contraindication to transjugular intrahepatic portosystemic shunt placement for refractory ascites. Clin Transl Gastroenterol 2021 Aug 1;12(8):e00378.

[40] Sultanik P, Lherault G, Bouzbib C, et al. Prevalence and prognosis of patients with MASLD-related cirrhosis after an ICU hospitalization in France: a single-centre prospective study. Aliment Pharmacol Ther 2024 Sep;60(6):796–810.

[41] Sultanik P, Kheloufi L, Leproux A, et al. Other causes of neurocognitive impairment than covert hepatic encephalopathy (CHE) are very frequent, either alone or associated with CHE, in cirrhotic patients with cognitive complaints. Aliment Pharmacol Ther 2024 Sep;60(6):749–764.

Keywords: Overt hepatic encephalopathy; cirrhosis; TIPS.

Received 16 January 2025; received in revised form 23 July 2025; accepted 4 August 2025; Available online 11 August 2025

Journal of Hepatology, Volume *7*

Supplemental information

Overt hepatic encephalopathy after elective and preemptive TIPS: Risk factors and prognosis

Marika Rudler, Charlotte Bouzbib, Philippe Sultanik, Charles Roux, Paul Primard, Mélisande Jorus, Lyes Kheloufi, Nicolas Weiss, Asier Rabasco Meneghetti, Benjamin Poussot, Hélène Larrue, Christophe Bureau, José Ursic Bedoya, Sarah Mouri, and Dominique Thabut

Overt hepatic encephalopathy after elective or preemptive TIPS: risk factors and prognosis

Marika Rudler, Charlotte Bouzbib, Philippe Sultanik, Charles Roux, Paul Primard, Mélisande Jorus, Lyes Kheloufi, Nicolas Weiss, Asier Rabasco Meneghetti, Benjamin Poussot, Hélène Larrue, Christophe Bureau, José Ursic Bedoya, Sarah Mouri, Dominique Thabut

Table of contents

Fig. S1…………………………………………………………………………………2

Fig. S2…………………………………………………………………………………2

Fig. S3…………………………………………………………………………………3

Fig. S4…………………………………………………………………………………3

Table S1………………………………………………………………………………...4

Table S2………………………………………………………………………………...5

Table S3………………………………………………………………………………...6

Table S4………………………………………………………………………………...8

Table S5……………………………………………………………………………….10

Table S6……………………………………………………………………………….10

Fig. S1.

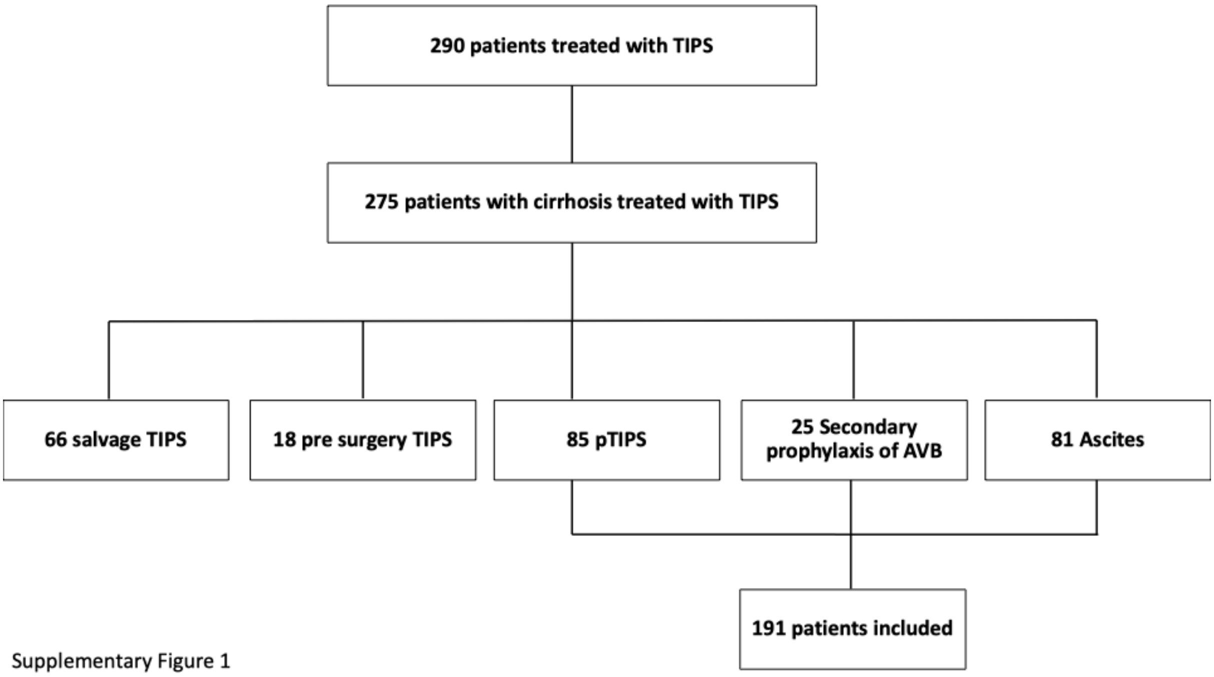

Supplementary Figure 1

Fig. S2.

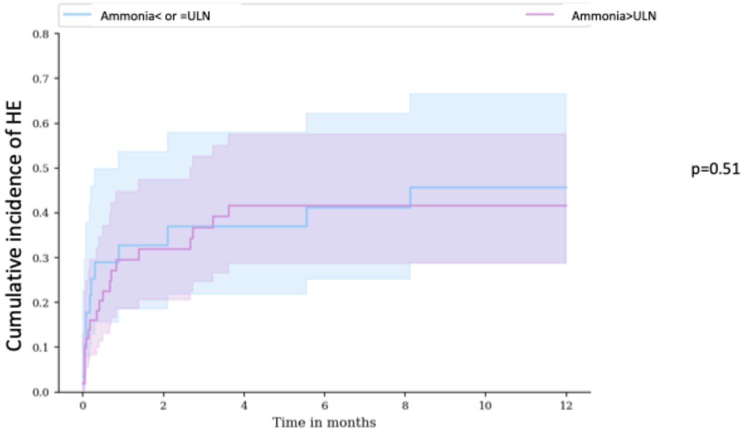

		0	2	4	6	8	10	12
Ammonia< or =ULN	No							
	At risk	27	16	15	14	13	11	0
	Censored	0	3	3	3	4	5	16
	Events	1	9	10	11	11	12	12
Ammonia>ULN	Yes							
	At risk	49	28	22	21	19	18	0
	Censored	1	8	10	11	13	14	32
	Events	1	15	19	19	19	19	19

Supplementary Figure 2

Fig. S3.

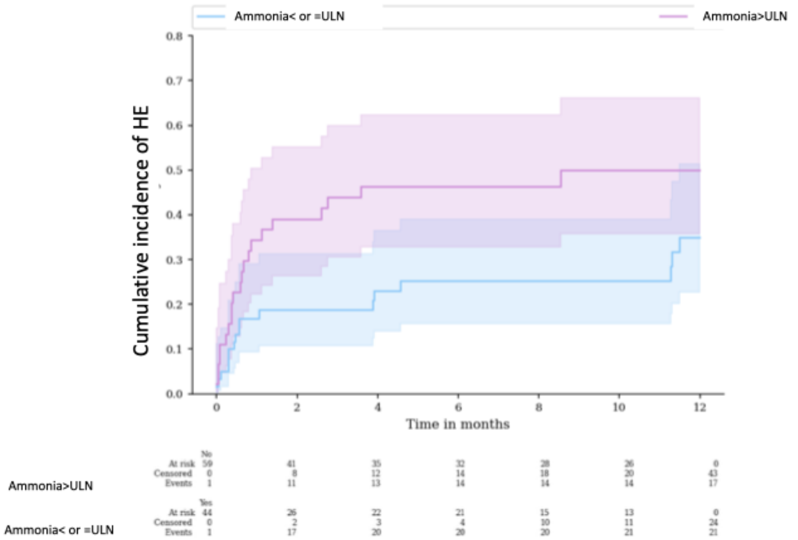

p=0.04

Supplementary Figure 3

Fig. S4.

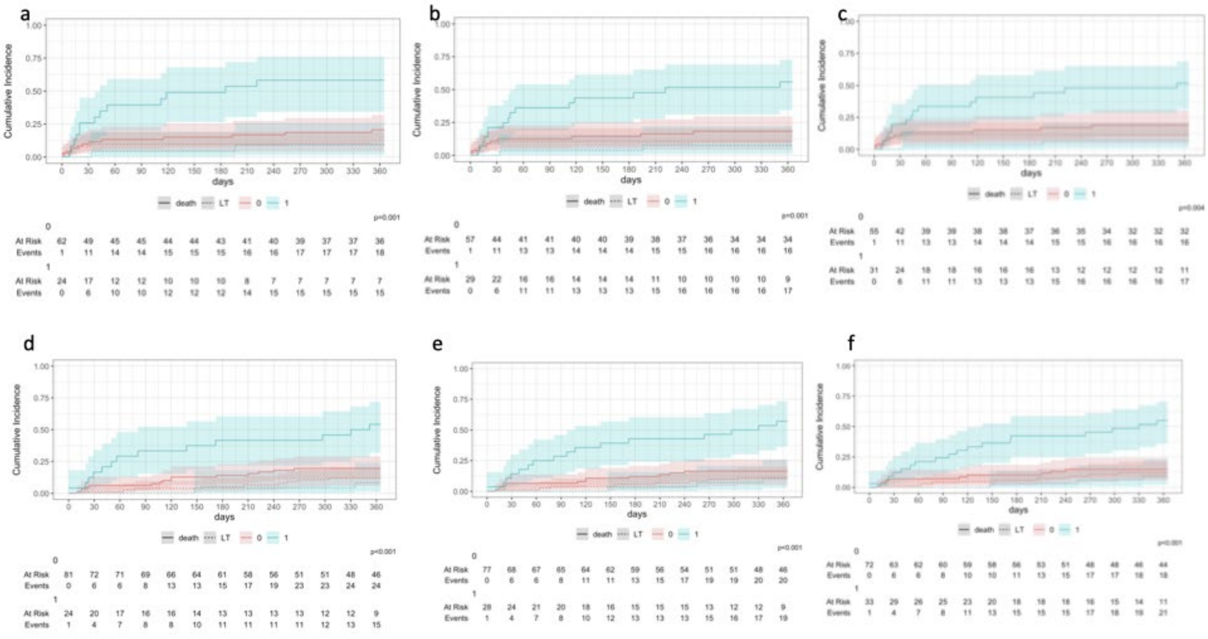

Supplementary Figure 4

Table S1: Baseline Characteristics of patients treated with elective TIPS according to TIPS indication

Variable	Ascites/hydrothorax (n=80)	AVB/surgery (n=26)	p value
Age (years)	59 (57-61)	60 (56-64)	0.82
**Male Gender, n (%)**	63 (78.7)	20 (76.9)	0.99
**BMI (kg/m^2)**	26 (23-30)	25 (23-29)	0.25
**Obesity/overweight (%)**	48 (60)	13 (48)	0.44
**Type 2 diabetes, n (%)**	30 (38)	10 (38)	0.99
**Previous cardiac disease, n (%)**	17 (21.8)	5 (19.2)	0.99
**Previous OHE, n (%)**	18 (22.8)	5 (19.2)	0.80
**Child-Pugh score**	8 (7-10)	7 (5-11)	0.06
**MELD score**	12 (7-18)	11 (8-16)	0.40
**INR**	1.8 (1.6-2.1)	1.7 (1.5-2.2)	0.81
**Serum sodium (mmol/L)**	133 (128-142)	137 (130-144)	<0.001
**Albumin (g/L)**	31 (29-32)	28 (26-29)	0.01
**Bilirubin (μmol/L)**	22 (8-75)	23 (7-72)	0.93
**Ammonia (μmol/L)**	52 (20-180)	59 (16-130)	0.23
**HVPG (mmHg)**	16 (10-25)	17 (11-26)	0.68
**PPG (mmHg)**	7 (6-9)	6 (5-9)	0.47

Group comparisons of categorical variables were performed using Chi-squared test. A p value <0.05 was considered significant.
BMI, body mass index; OHE, overt hepatic encephalopathy; MELD, Model for End-Stage Liver Disease; INR, international normalized ratio; HVPG, hepatic venous pressure gradient; PPG, portal pressure gradient

Table S2: baseline characteristics of patients treated with elective TIPS in the validation cohort and in the study cohort

Variable	Cohort of validation (n=132)	Study cohort (n=106)	p value
Age (years)	59±9	60±9	0.89
**Male Gender, n (%)**	111 (84.1)	26 (78.3)	0.33
**BMI (kg/m^2)**	25±6	26±6	0.25
**Previous OHE, n (%)**	21 (15.9)	23 (21.7)	0.33
**Child-Pugh score**	8±1	8±1	0.10
**MELD score**	13±4	12±3	0.18
**INR**	1.7±0.3	1.4±0.2	0.24
**Serum sodium (mmol/L)**	134±5	135±4	0.46
**Albumin (g/L)**	32±7	30±5	0.04
**Bilirubin (μmol/L)**	30±28	23±16	0.06
**HVPG (mmHg)**	16±5	16±4	0.93
**PPG (mmHg)**	7±3	7±3	0.85

Group comparisons of categorical variables were performed using Chi-squared test. A p value <0.05 was considered significant.
BMI, body mass index; OHE, overt hepatic encephalopathy; MELD, model for end stage liver disease; INR, international normalized ratio; HVPG, hepatic venous pressure gradient; PPG, portal pressure gradient.

Table S3: comparison of clinical, biological and hemodynamic characteristics of patients with or without OHE occurrence after pTIPS

Variable	No OHE (n=45)	OHE (n=39)	p value
Age (years)	52 (46-60)	54 (45-63)	0.54
**Male Gender, n (%)**	35 (78)	36 (92)	0.08
**BMI (kg/m^2)**	26 (23-30)	24 (22-29)	0.21
**Cause of liver disease n, (%)**			
**Other**	7 (16)	7 (18)	0.19
**MASLD**	2 (4)	0 (0)	
**ALD**	23 (51)	15 (38)	
**MetADL**	13 (29)	17 (44)	
**Obesity/overweight (%)**	27 (60)	19 (48)	0.41
**Type 2 diabetes, n (%)**	5 (11)	9 (23)	0.15
**Previous cardiac disease, n (%)**	3 (7)	8 (20)	<0.10
**Previous OHE, n (%)**	8 (18)	10 (25)	0.43
**Child-Pugh Class, n (%)**			
**A**	3 (7)	3 (8)	0.40
**B**	7 (16)	11 (28)	
**C**	34 (77)	25 (64)	
**MELD score**	19 (16-23)	18 (15-23)	0.41
**Platelets count (G/L)**	88 (61-137)	78 (61-104)	0.29
**Hemoglobin (g/dL)**	8.1 (7.3-9.5)	8.4 (7.5-9.1)	0.68
**Lactate**	1.7 (1.4-2.1)	1.6 (1.3-2.1)	0.60
**INR**	1.8 (1.6-2.1)	1.7 (1.5-2.2)	0.81
**Fibrinogen (g/L)**	2.1 (1.8-2.4)	2.1 (1.9-2.3)	0.94
**Serum sodium (mmol/L)**	137 (133-138)	137 (132-139)	0.92
**Albumin (g/L)**	26 (24-29)	26 (23-29)	0.64
**Bilirubin (μmol/L)**	72 (47-129)	48 (30-122)	0.14
**Ammonia (μmol/L)**	60 (45-88)	70 (39-108)	0.76
**HVPG (mmHg)**	14 (13-21)	17 (13-18)	0.66
**PPG (mmHg)**	7 (6-9)	6 (5-9)	0.18
**Decrease of mean PPG gradient (%)**	50 (36-64)	54 (42-64)	0.46
**TIPS diameter**	10 (8-10)	10 (8-10)	0.70
**TIPS dilatation**	10 (8-10)	10 (8-10)	0.85

Group comparisons of categorical variables were performed using Chi-squared test. A p value <0.05 was considered significant.
BMI, body mass index; MASLD, Metabolic-dysfunction Associated Steatotic Liver Disease; ALD, Alcohol related liver disease; MetALD, Metabolic and Alcohol related Liver Disease; OHE, overt hepatic encephalopathy; HCC, hepatocellular carcinoma; AVB, acute variceal bleeding; MELD, Model For End-Stage Liver Disease; PT, prothrombin time ratio; INR, international normalized ratio; AST aspartate aminotransferase; ALT, alanine aminotransferase; NT-proBNP, N-terminal pro b-type natriuretic peptide; Clif-C-ACLF

Table S4: comparison of clinical, biological and hemodynamic characteristics of patients with or without OHE occurrence after elective TIPS

Variable	No OHE (n=65)	OHE (n=41)	p value
Age (years)	60 (52-65)	62 (54-67)	0.36
**Male Gender, n (%)**	52 (80)	31 (76)	0.63
**BMI (kg/m²)**	25 (22-27)	25 (21-30)	0.48
**Cause of liver disease n, (%)**			
**Other**	12 (19)	9 (22)	0.26
**MASLD**	7 (11)	9 (22)	
**ALD**	27 (41)	14 (34)	
**MetADL**	19 (29)	9 (22)	
**Obesity/overweight (%)**	36 (56)	24 (59)	0.98
**Type 2 diabetes, n (%)**	**19 (30)**	**21 (52)**	**0.04**
**Previous cardiac disease, n (%)**	**6 (10)**	**16 (39)**	**<0.001**
**Ischemic cardiopathy, n (%)**	**2 (3)**	**7 (17)**	**0.03**
**Atrial fibrillation, n (%)**	**1 (2)**	**5 (12)**	**0.03**
**Previous OHE, n (%)**	16 (25)	7 (17)	0.47
**Child-Pugh Class, n (%)**			
**A**	6 (9)	3 (7)	0.93
**B**	53 (83)	34 (83)	
**C**	5 (8)	4 (10)	
**MELD score**	11 (10-14)	12 (10-15)	0.34
**Platelets count (G/L)**	**124 (86-168)**	**95 (67-141)**	**0.04**
**Hemoglobin (g/dL)**	9.8 (8.5-11.5)	9.7 (8.4-12.4)	0.40
**Lactate**	1.3 (1.2-1.5)	1.3 (1.2-1.5)	0.98
**INR**	1.5 (1.1-1.8)	1.6 (1.4-1.9)	0.22
**Fibrinogen (g/L)**	3.2 (2.4-3.9)	3.0 (2.2-3.3)	0.17
**Serum sodium (mmol/L)**	136 (131-138)	134 (132-138)	0.97
**Albumin (g/L)**	**32 (28-34)**	**28 (25-31)**	**0.02**
**Bilirubin (µmol/L)**	17 (11-27)	22 (11-33)	0.23
**Ammonia (µmol/L)**	**40 (28-60)**	**55 (37-96)**	**0.01**
**Rifaximin n %**	**39 (61)**	**15 (36)**	**0.02**
**HVPG (mmHg)**	16 (14-19)	16 (14-20)	0.72
**PPG (mmHg)**	6 (5-8)	7 (4-8)	0.84

**Decrease of mean PPG gradient (%)**	58 (50-67)	63 (53-71)	0.38
**TIPS diameter**	8 (8-10)	10 (8-10)	0.08
**TIPS dilatation**	8 (8-10)	8 (8-10)	0.21

Group comparisons of categorical variables were performed using Chi-squared test. A p value <0.05 was considered significant.
BMI, body mass index; MASLD, Metabolic-dysfunction Associated Steatotic Liver Disease; ALD, Alcohol related liver disease; MetALD, Metabolic and Alcohol related Liver Disease; OHE, overt hepatic encephalopathy; HCC, hepatocellular carcinoma; AVB, acute variceal bleeding; MELD, Model For End-Stage Liver Disease; PT, prothrombin time ratio; INR, international normalized ratio; AST aspartate aminotransferase; ALT, alanine aminotransferase; NT-proBNP, N-terminal pro b-type natriuretic peptide; Clif-C-ACLF

Table S5: Univariate and multivariate analysis of factors associated with OHE after elective TIPS in the validation cohort

	Univariate analysis for OHE in the elective TIPS group (validation cohort) HR [95% CI], p-value	p	Multivariate analysis for OHE in the elective TIPS group (validation cohort) HR [95% CI], p-value	p
**Age**	1.03 [1.00-1.06]	0.04	1.03 [0.99-1.07]	0.07
**Previous OHE**	1.93 [0.98-3.80]	0.06	1.91 [0.95-3.82]	0.07
**Platelets count**	1.00 [0.99-1.00]	0.17		
**Serum sodium**	0.97 [0.92-1.02]	0.23		
**Albumin**	0.96 [0.91-1.01]	0.09	0.97 [0.92-1.02]	0.23
**Creatinine**	**1.01 [1.00-1.01]**	**0.03**	**1.01 [1.00-1.01]**	**0.04**
**MELD score**	1.03 [0.97- 1.10]	0.35		
**PPG**	0.98 [0.90-1.07]	0.67		

Bolded values indicate statistically significant differences (p < 0.05) (Fine and Gray models).

OHE, overt hepatic encephalopathy; MELD, model for end stage liver disease; PPG, portal pressure gradient.

Table S6: outcomes of patients treated with elective TIPS according to TIPS indication

Outcome	Ascites/hydrothorax (n=80)	AVB/surgery (n=26)	p value
**Death n (%)**	**29 (36.2)**	**6 (23.0)**	**0.24**
**LT**	**16 (20.0)**	**2 (7.7)**	**0.23**
**OHE**	**31 (38.8)**	**10 (38.5)**	**0.99**

LT, liver transplantation; OHE, overt hepatic encephalopathy;
